# Spatial‐Wavelength Multiplexing Error‐Controlled Photonic Analog Computing System

**DOI:** 10.1002/advs.202515689

**Published:** 2026-03-13

**Authors:** Tao Zhu, Bowen Zhu, Shicheng Zhang, Keren Li, Xianchen Wu, Yazhi Pi, Jie Yan, Daigao Chen, Bingli Guo, Xi Xiao, Lei Wang, Xiaochuan Xu, Xuwei Xue, Shanguo Huang, Zizheng Cao, Shaohua Yu

**Affiliations:** ^1^ Pengcheng Laboratory Shenzhen China; ^2^ School of Integrated Circuits Harbin Institute of Technology(Shenzhen) Shenzhen China; ^3^ School of Electronic Engineering Beijing University of Posts and Telecommunications Beijing China; ^4^ College of Physics and Optoelectronic Engineering Shenzhen University Shenzhen China; ^5^ National Optoelectronics Innovation Center Wuhan China; ^6^ College of Integrated Circuits Zhejiang University Hangzhou China

**Keywords:** optical analog computing, silicon photonic chip, reliable computing, microwave photonics

## Abstract

In the “post‐Moore era,” the growing challenges in traditional digital computing have driven renewed interest in analog computing. Photonic analog computing has emerged as an effective paradigm for overcoming the fundamental bottlenecks that constrain conventional analog accelerators, especially suited for high‐speed signal processing and next‐generation 5G/6G RF systems. However, existing photonic analog computing frameworks lack the flexibility required to accommodate diverse application scenarios. To address these challenges, a novel silicon photonic chip is proposed in this paper that leverages fully optical analog computation. This system features multichannel architectures to enable spatial multiplexing, a parallel array of four reconfigurable microring resonators performs wavelength division multiplexed differentiation computing. In addition, an FPGA‐based error correction algorithm is developed to monitor processing operations in real time, ensuring the fidelity of computational results. Experimental demonstrations show the system's capability to solve ordinary differential equations and its applications in signal generation, coherent fiber communications, microwave photonics, and image feature detection. Taking into account the spectral utilization efficiency across the entire C‐band, the theoretical on‐chip processing capacity of single unit is evaluated to reach up to 2.725 tera operations per second, providing a novel hardware framework and innovative directions for photonic analog computing.

## Graphical abstract

A novel photonic integrated circuit prototype implementing the concept of general‐purpose analog computing and demonstrate its capability in radio frequency applications. The chip features a multichannel architecture and performs fully optical analog computation with frequency‐domain parallel processing. An FPGA‐based error‐correction algorithm aims to eliminate thermally induced drift in the MRR core unit, and ensures the reliability of the processing operations and results.

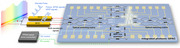

## 1 Introduction

Analog computing was once widely used in the mid‐20th century for solving differential equations and simulating dynamic systems. However, due to challenges like high noise sensitivity, unstable components, and limited scalability, it was overshadowed by digital computing. With semiconductor processes approaching their physical limits and digital architectures facing energy and performance challenges, the “post‐Moore era” has reignited interest in alternative computing methods. Analog‐like solutions, such as biological circuit, optical computing, and quantum computing, are gaining attention for their potential to bypass the von Neumann bottleneck and reduce digital logic power consumption [1, 2, 3]. Nevertheless, constrained by hardware footprint and power consumption limitations, along with inadequate computational function, reliability, and immature error‐correction methodologies, these emerging options have not gained widespread adoption.

Leveraging inherent physical advantages, photonic analog computing is emerging as a promising avenue to overcome current bottlenecks in analog computing. With advancements in semiconductor fabrication processes, photonic integrated circuits—particularly programmable photonic integrated circuits (PICs)—have evolved into an established platform for optical signal processing. Programmable PICs offer a highly parallel, energy‐efficient, and fast solution for a broad range of computational tasks [4, 5, 6], with inherent advantages, including low signal loss, ultra‐low latency, and strong electromagnetic interference (EMI) immunity during processing. Programmable PICs typically consist of reconfigurable networks formed by optical waveguides interconnected via 2×2 Mach–Zehnder interferometer (MZI) optical switches in specific topologies, such as rectangular [7, 8] and triangular [9, 10] configurations. These circuits support a variety of functions and have found applications in fields such as analog computing [11, 12, 13], differential equations solver [14, 15, 16], microwave photonics (MWP) [17, 18, 19, 20], quantum information processing [21, 22], matrix multiplexing [23], and photonic neural networks [24, 25, 26, 27, 28]. Complementing electronic controllers, photonic analog computing chips based on PIC technology leverage their distinct advantages in high‐bandwidth, ultra‐low power processing to enable on‐chip all‐optical signal handling. This approach is particularly suitable for high‐speed signal processing and next‐generation radio frequency (RF) systems, including 5G/6G applications.

However, existing photonic analog computing chips lack adaptable functionality for RF applications like microwave photonics and optical communications. Conventional photonic analog computing frameworks typically employ single‐channel configurations or exhibit only limited parallel processing capacity. Although such architectures suffice for validating basic differentiation and integration operations, they prove inadequate for high‐dimensional signals processing or large‐scale datasets, thereby restricting the universality of application scenarios for the photonic analog computing chip [13, 29, 30]. To address diverse RF‐photonic applications—encompassing communication, computation, and detection—novel photonic analog computing chip architectures with advanced processing frameworks are imperative. As early as 1941, Shannon introduced the General‐Purpose Analog Computer (GPAC) concept, demonstrating that ordinary differential equations (ODEs) could be mapped to analog circuits using integrators, adders, and multipliers [31, 32]. Consequently, exploring ways to combine the inherent continuity nature of analog computing with configurable multi‐functionality and reliable computing within the system framework constitutes a critical challenge for deploying photonic analog computing chips in future photonic RF systems. In addition, silicon photonic chips are particularly sensitive to temperature variations, and the dense arrangement of components intended to minimize chip size exacerbates thermal crosstalk, impacting the optical phase of on‐chip signals. Thus, developing error‐correction capabilities for PICs has become a crucial research focus [33, 34], aiming to ensure robust and reliable performance in practical applications.

In this paper, we propose a silicon photonic chip that realizes a fully optical and reconfigurable analog computing inspired by the GPAC concept. The system features an innovative four‐channel architecture comprising two optical switching matrices coupled with a parallel of four reconfigurable microring resonators (MRRs). The optical switching matrix enables on‐chip signal splitting, combining, and routing. As the core processing unit, MRRs have capable of performing analog differentiation operations and being configured as versatile optical filters. It also facilitates frequency‐domain parallel wavelength‐division multiplexing (WDM) signal processing on one structure, significantly boosting the system's processing capacity estimated to exceed 2.7 TOPS. This four channels architecture offers two key advantages: It enables multi‐dimensional signal processing, enhances computational throughput and supports flexible computational functionality through topological arrangements, exhibiting a general‐purpose capability across diverse scenarios. It also facilitates a processing‐in‐memory architectures, overcoming the von Neumann bottleneck by optical computing and transmitting/storing results optically, thereby avoiding losses and inefficiencies inherent in optoelectronic conversion. Additionally, an FPGA‐based feedback loop corrects thermal drift and crosstalk in operating MRRs, addressing a persistent challenge in photonic computing and enhancing system robustness and computational fidelity. Through extensive experiments—ranging from solving ordinary differential equations (ODEs) and generating ultra‐wideband (UWB) signals to demodulating WDM binary phase‐shift keying (BPSK) data and high‐speed image edge detection—we show that integrated photonic analog computing chip can be both robust and broadly applicable under various configurations. This computing framework provides an energy‐efficient, scalable analog computing solution for next‐generation communication, MWP and RF applications.

## 2 System Design and Characterization

### 2.1 Principle of Spatial‐Wavelength Multiplexing

The GPAC is a theoretical framework that models analog computation through a network of interconnected components, and the primary components include adders, multipliers, integrators, and differentiators. These components are specifically designed to perform fundamental mathematical operations, which enables the simulation of a wide range of dynamic systems. The GPAC model is particularly adept at solving systems of ODEs, making it highly suitable for modeling physical phenomena in fields such as physics and engineering. Shannon's work established that the functions computable by the GPAC are precisely those that are differentially algebraic [31, 35]. A function $f:R\to R^{k}$ is generated by a GPAC if and only if all of its components satisfy a polynomial differential equation of the form:

(1) $$p\left(t,y,y^{\prime },\cdots ,y^{\left(n\right)}\right)=0,$$

where p is a nonzero polynomial with real coefficients, and n∈N. Inspired by the principles of GPAC, we propose an innovative PIC that enables all‐optical analog computation with real‐time error correction.

**FIGURE 1:**
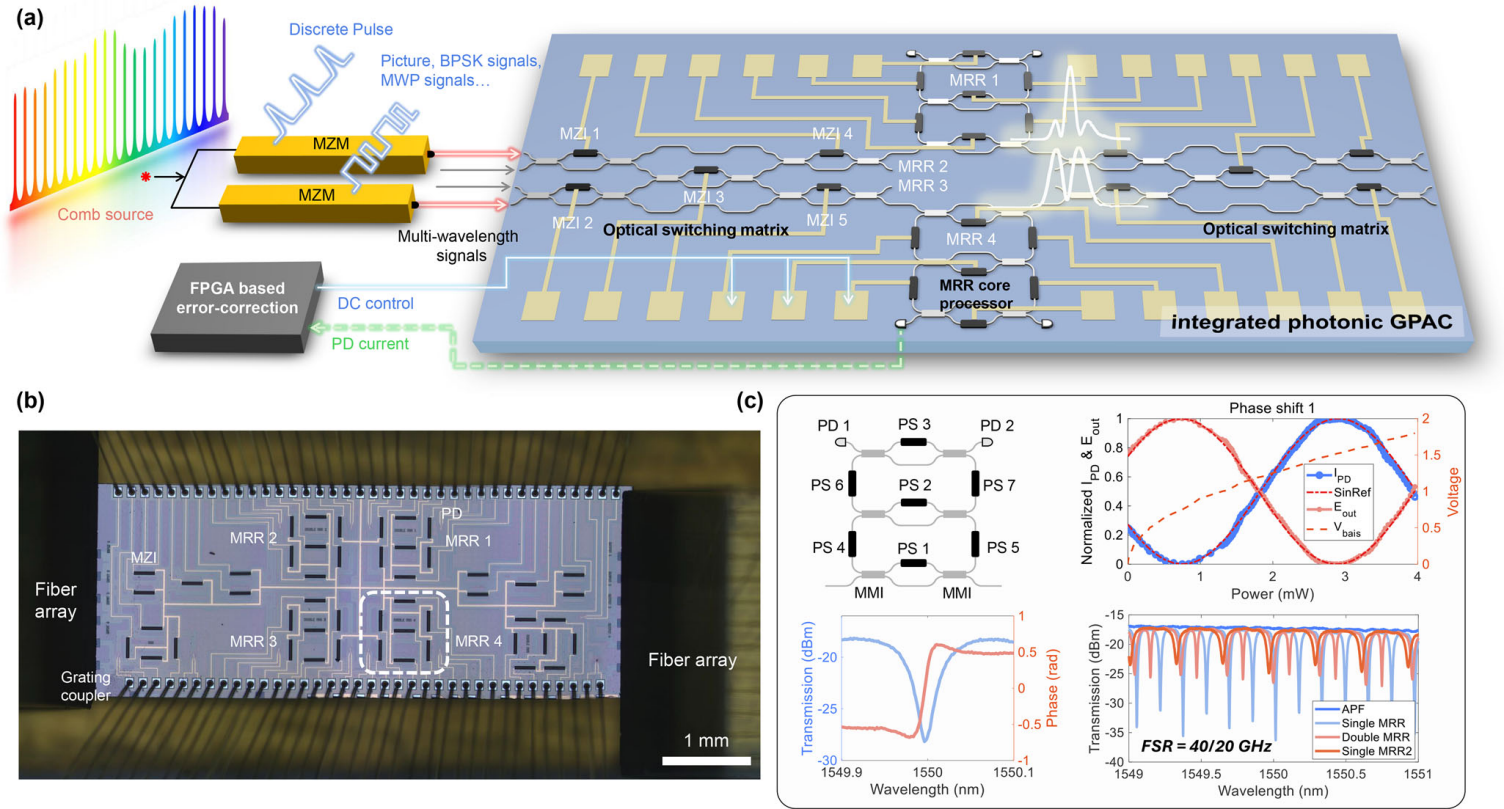
Chip design of GPAC prototype framework. (a). Schematic diagram of spatial‐wavelength multiplexing reliable photonic analog computing system. (b). Microscopic image of the integrated silicon photonic circuit. Note that the optical micrograph includes additional isolated single‐ring and dual‐ring MRR test structures used for independent device characterization. (c). A detailed structural diagram of the MRR core processors. PS: phase shifter, MMI: multi‐mode interference. The right panel of the figure presents the fundamental characterization results of the micro‐ring resonator (MRR). By applying a voltage to phase shifter 1 (PS1), the optical power coupled into the micro‐ring can be precisely tuned. Results demonstrate an inverse relationship between the photodetector (PD) current and the optical power at the Through port, with both parameters exhibiting a sinusoidal dependence on the phase shifter's power. The orange dashed line represents the voltage applied to PS1, where the square of the voltage is proportional to power. The lower‐left image in the figure presents the simulated spectral response of the MRR. When the optical power output at the Through port reaches its minimum, a π‐phase shift occurs, which is a critical feature enabling the use of MRRs for differentiation operations. The lower‐right image displays the experimentally measured spectral response of the MRR. By adjusting the voltage applied to the phase shifter (PS), the MRR can be reconfigured into various operational modes, including an all‐pass filter (APF), a single‐ring structure, and a double‐ring structure, with FSRs of 40 and 20 GHz, respectively.

The PIC features four parallel channels and reconfigurable MRRs, as shown in Figure 1a. The architecture consists of three primary components, two optical switching matrices and four processing core units in the middle. These three parts are connected in sequence, and the grating couplers are adopted at the input and output ports for the coupling of optical signals. The optical switching matrix is composed of five MZI optical switches through topological cascading, which enable the routing, switching, and merging of optical signals across four channels on the chip. Each MZI switch consists of two mode multiplexing interferometers (MMI) and a thermal phase shifter (PS). These switches can operate in three distinct states: bar state, cross state, and tunable coupler. The four‐channel optical carrier signals, which have been modulated to carry data information, enter the processing unit from the input ports on the left. The input optical signals can be represented as a 4×1 vector. Each channel of mixed optical signals can simultaneously contain wavelengths ranging from $\lambda_{1}$ to $\lambda_{4}$. After propagating through the 4×4 switching matrix, optical signals redistribution operations can be performed across all wavelengths simultaneously, achieving the spatial‐frequency interleaved computation paradigm.

Each of the four channels features a core processing unit, which consists of a reconfigurable double ring structure, as shown in Figure 1c. The MRRs regulate the coupling of optical signals via three tunable MZI switch structures, allowing for dynamic adjustment of optical properties. Specifically, the system supports two distinct free spectral ranges (FSR), specifically 40 and 20 GHz. This design enables 15 reconfigurable configuration states, including three types of all‐pass filters. Figure 1c illustrates four spectral responses of these states. Additionally, four thermally tunable phase shifters on both sides of the microring can be adjusted to shift the spectral response of the microring red or blue on the spectrum. The core processing unit can be configured as a time‐domain optical differentiator, enabling first‐order differentiation of input optical signals [36]. The frequency domain transfer function is given as $T_{\omega }=j\left(\omega -\omega_{0}\right)$, where $\omega_{0}$ represents the optical carrier frequency. Meanwhile, there are two integrated Ge photodetectors (PD) at each of the two drop ports of the microring, respectively. These PDs are utilized to detect the intensity of optical signals, enabling the state of the microrings to be locked and controlled through feedback.

**FIGURE 2:**
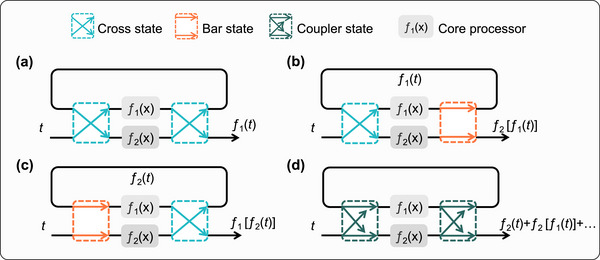
Schematic diagram of spatial multiplexing and multifunction configurations of chip.

By configuring the states of the optical switches, the proposed chip can be dynamically reconfigured to facilitate flexible analog computing operations and achieve complex computing functions, as illustrated in Figure 2. Suppose that the processing of the input optical signal x(t) by the core processing units of the two channels is denoted as $f_{1}\left(x\right)$ and $f_{2}\left(x\right)$, respectively. The simplest case is to perform the processing on the input optical signal only once, as shown in Figure 2a. In addition, the computation results can be transmitted back onto the chip through a loop back, constructing a second‐step computation, as shown in Figure 2b,c. Figure 2d involves a more complex situation. By configuring the MZI optical switches into the coupling state (tunable coupler, TC), the combining of on‐chip optical signals can be achieved, thus the output result is the sum of two computational results. This computational framework eliminates the energy penalties inherent in electro‐optic conversions, and forms a resonant structure similar to a MRR. When the input optical signal is a pulsed signal, the output of the system corresponds to the steady‐state solution of the input optical signal within the resonant structure. Therefore, it enables the implementation of calculations such as Taylor series expansion, as well as the solution of ODEs with constant coefficients.

### 2.2 Configuration for ODE Solving

**FIGURE 3:**
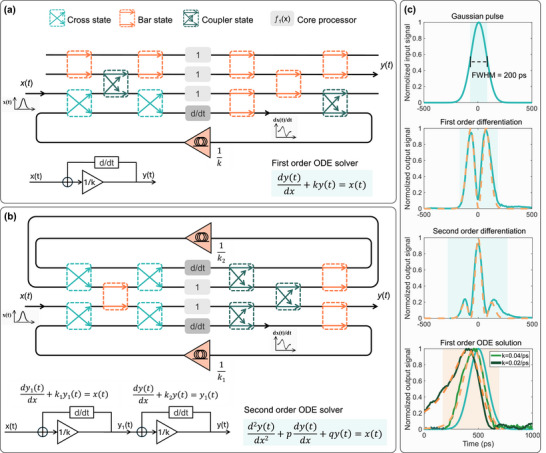
Schematic diagram of GPAC configured as ODE solver utilizing real time differential operations. (a) Configuration of a first‐order ODE solver. (b) Configuration of a second‐order ODE solver on a single chip. (c) Waveform results of first‐order and second‐order differentiation applied to a Gaussian pulse with an FWHM of 200 ps, and the first‐order ODE solutions with a Gaussian pulse as input x(t). The orange dashed line denotes the simulation results.

Shannon's GPAC produces solutions of ODEs as its output and serves as a distinguishing feature. The proposed chip leverages core processing units to perform differential operations, with configurable MZI optical switch and optical loopback interconnects enabling programmable ODE solutions, as illustrated in Figure 3a. The schematic of the solver system architecture is shown in the lower‐left corner of Figure 3a. The system propagates the input pulse signal x(t) into a feedback loop, where iterative signal processing ensures convergence to a stable state, and the output signal y(t) is the solution to the equation of $\frac{dy}{dx}+ky\left(t\right)=x\left(t\right)$. The d/dt block in the diagram represents the differentiation operation performed by a reconfigurable MRR, coefficient 1/k is adjusted via an erbium‐doped fiber amplifier (EDFA), while optical signal combining is achieved through on‐chip MZI optical switches. Upon achieving loop stabilization, the solution y(t) to the first‐order ODE is obtained. The detailed mathematical derivation of this process, including the formulation of the feedback dynamics and stability criteria, is provided in the Supporting Information. This approach leverages the inherent properties of the feedback architecture to enable real‐time analog computation of ODE solutions, offering a robust alternative to traditional numerical methods.

Within this solver system architecture, the fiber loop‐back configuration in ODE solver experiment can be treated as an integrated‐photonic recurrent processors (IPRPs) frameworks [37], which eliminates the repeated O/E/O conversions that typically dominate noise accumulation in cascaded analog systems, including thermal noise, quantization noise of the electrical devices, thereby significantly suppressing noise propagation and preserving signal integrity in multi‐stage computation. Figure 3c demonstrates the waveform results of first‐order and second‐order differentiation applied to a Gaussian pulse with a full width at half maximum (FWHM) of 200 ps. Based on the captured waveforms, the photonic processor accurately performs differentiation on the input signal, with the output showing excellent agreement with the simulation results. We also presents the solution of a first‐order ODE, as shown in the lower part of Figure 3c. When a Gaussian pulse (FWHM = 200 ps) is applied as the input x(t), the solver system produces the corresponding ODE solution y(t) for a known parameter 1/k. Under this condition, the GPAC system operates in a dynamically balanced state, and the acquired output waveform matches well with the simulation results. It should be noted that the system employs a pure direct‐detection optical readout which constrains the processor to real‐valued operations, while it is possible that future extensions using coherent detection or phase‐retrieval techniques could enable generalized complex‐valued processing.

Theoretically, the GPAC system can also be configured to solve second‐order ODEs by cascading two first‐order ODE blocks, where the optical output of the first stage directly drives the second, as shown in Figure 3b. Higher‐order ODEs can be realized by extending this cascading approach across multiple stages or multiple chips. More generally, any linear constant‐coefficient *N*th‐order ODE can be equivalently reformulated as an N‐dimensional first‐order linear system. This mathematical equivalence establishes the architectural feasibility of implementing higher‐order ODE solvers using multiple first‐order computational primitives arranged in a cascaded or modular manner. Such cascading strategy has been widely adopted in prior photonic implementations of differential and difference‐equation solvers based on microrings, microcavities, and semiconductor optical amplifiers, demonstrating both device‐level feasibility and coefficient tunability for first‐ and higher‐order systems [38, 39]. In practice, scaling to higher‐order systems will be constrained by several physical factors, including accumulated optical loss, amplifier ASE noise, and stability of the feedback loops, particularly when optical amplification is employed. To address these factors, we experimentally characterized the noise behavior and stability of the EDFA operating within a feedback loop, providing empirical insight into the dominant physical limitations (See Figure S4). Experimental measurements indicated that when the system operated stably, the low‐noise EDFA in the loop‐back configuration did not induce significant oscillation. Also, fabrication‐induced variations and packaging loss can further limit the achievable orders in deep cascades. As a result, higher‐order ODE solving is feasible only within a bounded number of stages. We further present higher order ODE solving demonstrations (See Figures S6–S8), realized by using the first‐order solutions as the equivalent input of the same first‐order solver. These demonstrations are intended to verify the fundamental feasibility of higher‐order cascading and the performance of the current packaged chip under realistic noise and loss conditions. The current GPAC architecture focus on practically relevant operating regimes such as RF signal processing, microwave photonics, and optical communication applications, whereas deeper cascading and fully integrated higher‐order ODE solving represents an important direction for future experimental investigation.

### 2.3 FPGA‐Based Real‐Time Correction

**FIGURE 4:**
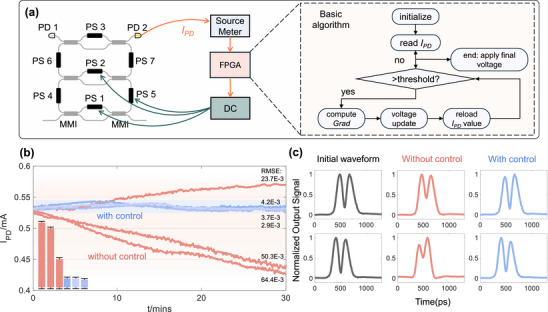
FPGA‐based real‐time error‐correction. (a) Schematic diagram of FPGA‐based real‐time error‐correction system and basic algorithm. (b) Recorded PD current values for 30 min with/without error‐correction algorithm. (c) Oscilloscope records the time‐domain waveform changes of the first‐order differential result of a Gaussian pulse with/without error‐correction algorithm after 30 min.

Enhancing the reliability of computational results is a critical challenge in analog computing, where the credibility of the process depends fundamentally on the stability of the hardware. As is well known, silicon photonic chips are susceptible to temperature variations—stemming from both environmental fluctuations and on‐chip thermal crosstalk—as well as fabrication imperfections, which can induce significant drift in the operational state of the micro‐ring resonators. To address this, we implemented a real‐time, closed‐loop error correction mechanism, with the control architecture illustrated in Figure 4a. The core processing unit incorporates an on‐chip PD that continuously monitors the optical power at the drop port. This PD current serves as a direct indicator of the MRR's coupling condition; a maximum current corresponds to the critical coupling state required for accurate differentiation. Leveraging this feedback, we developed a gradient descent algorithm hosted on a Field‐Programmable Gate Array (FPGA). The controller dynamically adjusts the voltages of the on‐chip thermal phase shifters to maximize the PD current, effectively locking the MRR's resonance wavelength against environmental drift. A detailed mathematical derivation and the hardware implementation of this algorithm are provided in the Supporting Information (See Figure S10).

To quantitatively evaluate the robustness, we conducted a statistical analysis of system stability over multiple trials. The quantitative performance metrics are summarized in Table 1. As shown in the PD current monitoring results in Figure 4b, in the absence of control, the system exhibited significant instability with large random fluctuations, resulting in a high average RMS deviation of 0.0461 mA. Notably, the deviation magnitude varied drastically between individual trials (ranging from $23.7\times 10^{-3}$ mA to $64.4\times 10^{-3}$ mA), highlighting the unpredictable stochastic nature of thermal drift. In contrast, the active control algorithm successfully suppressed these fluctuations. The average RMS deviation was reduced by approximately 12.8 times to 0.0036 mA. More importantly, the consistency observed across all three corrected trials (with deviations consistently kept below $4.2\times 10^{-3}$ mA) demonstrates the high reliability and reproducibility of the feedback loop.

**TABLE 1:** Quantitative performance summary of the photonic GPAC chip: System stability (PD current) and signal fidelity (differentiation waveform) over 30 min.

Category	Metric/parameter	Without control (drifted)	With control (corrected)	Improvement factor
I. System stability *(PD current RMSE)*	Individual trials	Trial 1: 50.3	Trial 1: 4.2	—
	($10^{-3}$ mA)	Trial 2: 23.7	Trial 2: 3.7	
		Trial 3: 64.4	Trial 3: 2.9	
	Mean RMS deviation ($I_{\mathrm{PD}}$)	0.0461 mA	0.0036 mA	∼12.8× (92.2%)
II. Signal fidelity *(Waveform quality)*	Waveform RMSE	0.0705	0.0208	∼3.4× (70.5%)
	Pearson correlation (*r*)	0.9448	0.9960	Near‐Ideal

**TABLE 2:** Benchmarking state‐of‐the‐art architectures for integrated analog computing systems: A comparative analysis of technical pathways.

	Platform	BW (GHz)	Core processor	Min pulse(ps)	Proc. speed (TOPS)	Accuracy & Robustness	Scalability	Tunability & Multi‐function
2016 [13]	Si	55	Tunable MRR	33	N/A	N/A	N/A	N/A
2018 [49]	Si	40	Reconf. grating	290	N/A	N/A	Yes	N/A
2020 [50]	Si	40	FPDA	85	N/A	N/A	Yes	Wavelength multiplexing
2021 [9]	Si	N/A	MZI (Reck)	N/A	N/A	High	Yes	complex‐valued ONC
2024 [51]	Si	N/A	DONN	N/A	10^16^ FLOPS	High	Yes	Static
2024 [52]	TFLN	60	MZI	N/A	0.12	N/A	N/A	Time‐division multiplexing
2024 [29]	TFLN	>67	MRR/MZI	9.6	N/A	N/A	N/A	N/A
2024 [53]	Si	N/A	Matasurface	N/A	N/A	High Fidelity	Yes	polarization‐spatial multiplexing
2024 [27]	Si	N/A	MZI (Clements)	100	0.59/inference	High	Yes	Forward‐only in situ training
2025 [16]	Si	<20	Tunable MRR	N/A	15.3	High	Yes	Parallel PDE solver
2025 [30]	Si	<20	MZM+TDL	25	N/A	N/A	N/A	Parallel processing
**This work**	Si	40	Tunable MRR	31	0.125/MRR (Demo) 2.725/MRR (Theoretical)	High (FPGA‐based real‐timecorrection)	Yes	Spatial‐wavelength multiplexing

N/A = Information not available; TDL = Tunable delay line; ONC = Optical neural chip; MRR = Microring resonator; MZI = Mach‐Zehnder interferometer; FPDA = Field‐programmable photonic device array; ODE = Ordinary differential equation; BW = Bandwidth; Reconf. = Reconfigurable; Samp. = Sampling; Proc. = Processing.

This stabilization of the operating point directly translated into superior signal fidelity in the analog computation. As shown in the waveform comparison in Figure 4c, while the uncorrected output exhibited significant distortion after 30 min, the corrected system maintained the precise first‐order differentiation waveform. Quantitatively, as detailed in Table 1, the system reduced the waveform RMS error (RMSE) by 70.5% (from 0.0705 to 0.0208) and maintained a Pearson correlation coefficient of r=0.9960, effectively preserving the accuracy of the analog computation against environmental disturbances. The long‐term evaluation of this scheme demonstrates that our on‐chip design, combined with the proactive correction algorithm, ensures optimal fidelity of the processed results and provides a fundamental safeguard for addressing reliability concerns in analog computing.

### 2.4 Demonstrations of Microwave Photonics and Communication Applications

**FIGURE 5:**
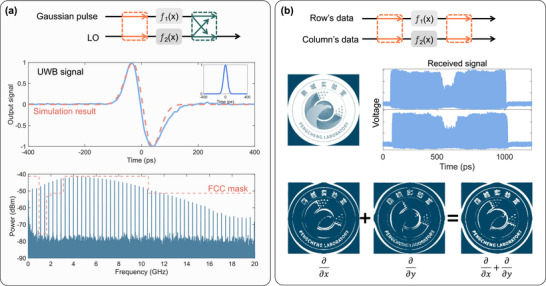
UWB signal generation and high‐speed images' edge features detection utilizing spatial multiplexing. (a) The upper portion illustrates the on‐chip configuration for UWB signal generation, the middle illustrates the time‐domain waveform of the output UWB signal, insert showing the input Gaussian pulse waveform. The lower portion displays the corresponding frequency spectrum of the synthesized UWB signal, with FCC mask. (b) The upper portion illustrates the on‐chip configuration for image edge detection, the middle shows the original picture and time‐domain waveforms of two received signals, the lower portion displayed the results of image edge features detection.

The proposed photonic analog computing chip is capable of addressing a broad spectrum of microwave–photonic and RF applications. We first experimentally demonstrated the generation of ultra‐wide band (UWB) signals based on differential operations, which provides a novel approach for the generation of UWB signals in the optical domain. UWB signals possess an extremely wide bandwidth and an extremely narrow signal pulse width. Under the same signal‐to‐noise ratio, they can offer a higher channel capacity, which makes them applicable to the rapid transmission of large amounts of data within short distances. Moreover, UWB signals can also conduct high‐resolution detection on targets at close ranges [40, 41, 42]. UWB signals can be accomplished by utilizing on‐chip combination approach, as shown in Figure 5a. A Gaussian pulse with an FWHM = 100 ps is introduced into one channel on the chip, while an optical signal of the same wavelength is fed into the other channel as the local oscillator signal. At this point, the core processing unit $f_{1}\left(x\right)$ performs a first‐order differential operation, and the other processing unit $f_{2}\left(x\right)$ does not perform any processing, which is equivalent to $f_{2}\left(x\right)=1$. The experimentally generated signal exhibits excellent agreement with the numerical simulation. In our experiment, the measured spectrum of the generated UWB signal is compared against the Federal Communications Commission (FCC) mask to verify spectral compliance. This comparison confirms that the photonic signal processing scheme can generate UWB waveforms with controlled spectral shaping, such that the signal energy remains within the permitted bandwidth and power levels.

Another application of GPAC chip is image edge detection, as shown in Figure 5b. Initially, the grayscale information of the image is extracted. The grayscale image is subsequently one‐dimensionalized in a row‐by‐row or column‐by‐column manner to generate continuous 1D image information. By performing differential processing on the image signal, those parts with drastic changes in grayscale values can be calculated, thus achieving edge detection. Thereafter, the computational results are reconstructed into a 2D image, which serves as the outcome of the image edge detection. However, this method suffers an issue that if the image is transformed row‐by‐row, the computational process cannot effectively detect the changes in grayscale values of the original image in the column direction. A promising solution is to one‐dimensionalize the image both row‐by‐row and column‐by‐column, and simultaneously perform differential calculations on the information in both directions. Finally, by combining the two results, the obtained detection outcome exhibits a better performance compared to the results of single calculation.

A notable advantage of this GPAC chip is its capability for parallel processing in the frequency domain, which allows it to significantly enhance processing efficiency in a highly compact, energy‐efficient, and cost‐effective manner. To verify this, we demonstrated the chip's ability to simultaneously demodulate WDM BPSK signals at the receiver end in high‐speed optical communication applications. In optical fiber communication, BPSK modulation offers the same spectral efficiency as On–Off Keying (OOK) but exhibits better tolerance to nonlinear distortions and higher receiver sensitivity. However, since the carrier is suppressed, demodulating BPSK signals requires complex coherent receivers or precisely controlled delay‐line interferometers (DLIs). By configuring MRRs as a temporal differentiator to replace the DLIs, differential detection of BPSK signals can be achieved [43, 44, 45]. In [46], the demodulation of a 10 Gbps BPSK signal using a MRR was demonstrated for the first time. Specific descriptions of demodulation principle and simulation results can be find in the Supporting Information. In our experiment, we showcased the MRR demodulation of single‐wavelength 32 Gbps BPSK signal and 5×25 Gbps WDM BPSK signals, validating the chip's application in optical communication and its capability for parallel processing in the frequency domain.

**FIGURE 6:**
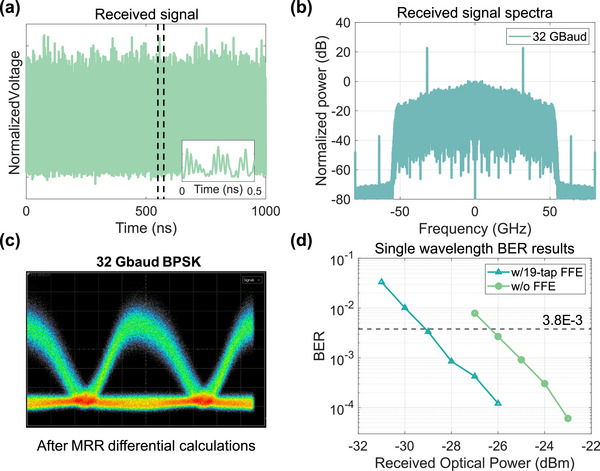
Demodulation of single‐wavelength 32 Gbaud BPSK signal. (a) Received demodulated BPSK signal and (b) its corresponding spectra diagram. (c) Eye diagram of the 32 GBaud BPSK signal after MRR differential calculation. (d) BER results with/without a simple 19‐tap linear equalizer.

**FIGURE 7:**
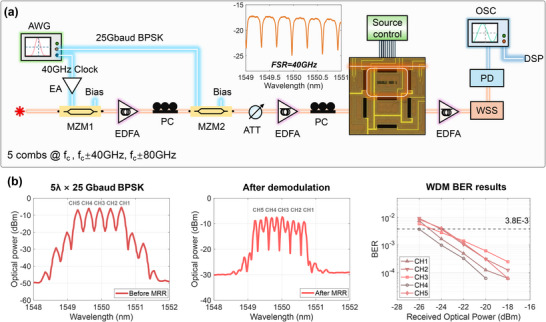
Demodulation of 5×25 Gbaud WDM BPSK signals. (a) Experimental setup, insert is the spectra response of the core processor MRR. EA: electrical amplifiers; PC: polarization controller; ATT: attenuator; DUT: device under test; WSS: wavelength selective switch; PD: photodetector; AWG: arbitrary waveform generator; OSC: oscilloscope. (b) Signal spectra before and after MRR processing and the BER results.

We first tested the demodulation of single‐wavelength 32 Gbaud BPSK signal to verify feasibility before conducting the WDM experiment. In this case, only one CW light source was modulated and fed into the second channel. Here, a processing unit of the PIC chip was activated, and its reconfigurable double ring structure was configured as a single MRR with an FSR of 40 GHz, enabling it to handle BPSK signals with sufficiently large bandwidths. Figure 6c displays the eye diagram of the signal after MRR processing, indicating that the coherent signals have been converted into a pulse sequence. Figure 6d shows the relationship between the bit error rate (BER) and the received optical power. After applying a simple 19‐tap linear equalizer, a receiver sensitivity of −29 dBm was achieved at the 7% HD‐FEC threshold. Figure 7a illustrates the experimental setup of WDM BPSK signal demodulation. For the WDM case, the first single‐drive MZM was driven by a 40 GHz clock to generate an optical frequency comb with intervals corresponding to the FSR of the processing MRR. After amplification, five lines of the frequency comb served as carriers and were fed into the MZM for simultaneous modulation, generating five 25 Gbaud WDM BPSK signals. These five signals were simultaneously demodulated by the MRR in channel two, as the case with a single wavelength. At the receiver, an optical attenuator (ATT) was used to adjust the received power. After processing the WDM BPSK signals through the MRR, a wavelength selective switch (WSS) selected the target wavelength signal, which was then converted into an electrical signal by a PD and captured by an oscilloscope. Figure 7b displays the signal spectra before and after MRR processing, with a resolution of 0.1 nm. We measured the BER results for each signal, as shown in Figure 7b. In the WDM case, the receiver sensitivity was approximately −24 dBm.

## 3 Discussion

Here, we first evaluate the chip's processing capabilities. The core processing units on the chip primarily rely on thermally tuned phase shifters to configure their operational states. Silicon‐based photonic chips offer a distinct advantage over other platforms, such as thin‐film lithium niobate (TFLN), in terms of relatively lower power consumption. As evidenced by the baseline test results in Figure 1c, the titanium nitride (TiN) heaters in the silicon photonic chip exhibit an efficiency of approximately 2.2 mW/π, whereas thermally tuned phase shifters in thin‐film ${\mathrm{LiNbO}}_{3}$ chips typically require hundreds of mW/π. Taking the BPSK signal demodulation as an example, when performing a 32 Gbaud single‐wavelength demodulation, only one core processing unit on the chip is in the working state. Under these conditions, the signal processing speed reaches 32 giga‐operations per second (GOPS). When the GPAC photonic chip performs demodulation of WDM BPSK signals by the same one MRR, it does not increase the power consumption of the chip. The reconfigurable MRR still operates in the state with a FSR of 40 GHz, yet the processing speed is multiplied. For instance, in the experiment validating the demodulation of 5 × 25 Gbps WDM BPSK signals, the processing speed of the chip in this experiment is 125 GOPS. Theoretically, if multi‐wavelength BPSK signals span the entire C‐band, 109 signals will be demodulated simultaneously. During the demodulation process, the chip can achieve a processing speed of 2.725 TOPS per MRR.

This value represents the maximum theoretical processing capability of a single MRR under ideal conditions. In practice, the measured spectral response of our on‐chip microrings also covers the entire C‐band, which provide approximately 110 usable WDM channels with 40 GHz FSR. When accounting for other practical factors such as optical insertion loss and cross talk, the packaged chip used in our experiments can achieves computational throughput of approximately 1.25 TOPS per MRR. The detailed estimation methodology and cross talk analysis are provided in the Supporting Information, along with an estimation of total energy consumption of the hole system.

Expanding on these signal processing capabilities, it is crucial to recognize that the fundamental operations performed by our chip hold potential implications for artificial intelligence paradigms. While the demonstrated image edge detection represents a fundamental signal processing operation, its significance extends remarkably into the domain of Scientific Machine Learning (SciML), particularly regarding the recently proposed physics‐informed neural networks (PINNs) [47, 48]. Unlike conventional data‐driven neural networks, PINN explicitly embed governing differential equations into the training objective, requiring repeated and accurate evaluation of first‐ and higher‐order derivatives of the network output with respect to its inputs. In this context, differentiation is not an auxiliary feature‐extraction step but a core operation required to enforce the governing physical laws. The proposed GPAC system leverages its intrinsic optical differentiation capability to directly accelerate PINN workflows rather than for conventional neural network training. From a Sobolev training perspective, PINNs constrain the solution space by enforcing consistency at both the function and derivative levels, which significantly improves convergence stability and sample efficiency for stiff or high‐frequency partial differential equations. By physically implementing the differential operator E(x)=dI(x)/dx in the optical domain, the proposed photonic processor enables high‐fidelity analog differentiation that benefits from the inherent precision of continuous‐time photonic signal processing. The resulting gradient‐informed signals are delivered directly to the digital backend, where an FPGA‐based calibration and error‐correction loop is employed to ensure overall computational accuracy and robustness.

Moreover, the proposed MRR‐based differentiator inherently acts as a tunable high‐pass filter, suppressing low‐frequency baselines while selectively amplifying local gradient information. This physics‐aligned preconditioning mitigates spectral bias in PINNs and allows the digital network to focus its representational capacity on enforcing physical constraints rather than implicitly learning derivatives. Together, these results indicate that photonic differentiation provides a natural and efficient pathway for accelerating PINNs, while remaining outside the scope of general‐purpose deep learning accelerators. Therefore, the presented reliable analog computing architecture offers a specialized hardware primitive for neural networks, where the optical front–end handles high‐bandwidth and high‐fidelity differential calculus, allowing the digital back‐end to focus on nonlinear function approximation. This hybrid paradigm significantly alleviates the computational burden on digital processors, paving the way for energy‐efficient scientific discovery. The above clarification and analysis provide a foundation for future system‐level implementations that integrate the proposed GPAC with digital PINN backends.

A comparative analysis of state‐of‐the‐art analog computing chips is summarized in Table 2. For photonic neural network systems, most implementations employ the SOI platform, which provides a relatively power‐efficient on‐chip solution. In contrast, silicon‐based devices are bandwidth‐constrained; consequently, these studies primarily focus on verifying the feasibility of the computational architecture and its advantages such as low‐latency operation, rather than emphasizing the bandwidth of the processed signals. In recent years, PIC based on heterogeneous integration platforms, such as thin‐film lithium niobate (TFLN), have been extensively investigated. These platforms leverage the intrinsic material advantages (e.g., high electro‐optic coefficients and low optical losses) to achieve significantly enhanced operational bandwidths. However, the critical challenge of computational reliability—particularly analog computing—remains unresolved in existing systems. Our proposed architecture using an FPGA‐based real‐time error‐correction algorithm, ensuring environmental robustness and long‐term stability. The multichannel parallel processing design of our chip enhances computational efficiency by enabling simultaneous wavelength‐ and space‐division multiplexing, thereby maintaining signal fidelity and anti‐interference capability.

## 4 Conclusion

In this work, we propose a novel PIC prototype implementing the concept of general‐purpose analog computing and demonstrate its capability in microwave photonics applications. The chip features a multichannel architecture and is capable of adjusting to various operational functions. The core processing units, based on reconfigurable MRR structures, are able to perform fully optical analog computation with frequency‐domain parallel processing. An FPGA‐based error‐correction algorithm aims to eliminate thermally induced drift in MRR caused by crosstalk and ensures the reliability of the processing operations and results. Long‐duration calibration procedures, validated through extensive testing, ensure optimal fidelity of the processed results and provide a fundamental safeguard against reliability concerns in analog computing.

We demonstrate a case of solving an ODE based on analog differential computing, showcasing the completeness and functionality of the system as a GPAC implementation. For RF applications, we present three case studies for the chip's capabilities in optical communications and microwave photonics, including all‐optical UWB signal generation, high‐speed images' edge features detection utilizing spatial multiplexing, and demodulation of 5×25 Gbps WDM BPSK signals. By exploiting the periodic frequency‐domain response of the core processing units, the chip enables parallel processing of multi‐wavelength signals, facilitating wavelength‐division multiplexing and significantly enhancing computational throughput and energy efficiency. Based on estimations, the chip has the ability to achieve a theoretical processing speed over 2.7 TOPS, offering a new architectural framework and innovative directions for next generation's RF photonics.

Moreover, we explore the potential relevance of the GPAC chip to scientific machine‐learning applications, especially in the context of physics‐informed neural networks, where differential operators constitute the dominant computational workload. We believe that this study contributes to the ongoing exploration of general‐purpose analog computing in the post‐Moore's law era and provides a foundation for future scalable GPAC‐like architectures.

## 5 Experimental Section

### 5.1 Characterization of the Photonic Chip

The integrated photonic chip was fabricated on an 8″ wafer using a commercial 220 nm silicon‐on‐insulator (SOI) platform, with a 3 μm thick BOX. The chip has a total size of 5.9×2.3 mm. A silicon strip waveguide featuring a width of 500 nm is patterned on a SiN hardmask using an ArF lithography scanner and SiN dry etch, and its propagation loss is lower than 1.5 dB/cm at a wavelength of 1550 nm. The length of each ring resonator in the core processor is 1.757 mm, each MMI coupler in the MZI structure has 0.3 dB insertion loss. The vertical grating coupler has a $10^{\circ }$ incident angle to maximum the coupling efficiency, and the insertion loss of the grating coupler is <4 dB at C‐band. On‐chip photodetectors are fabricated using a 500 nm thickness Ge with responsivity >0.8 A/W, the measured dark current of high‐speed PD is about 20 μA, and its O/E 3 dB bandwidth is larger than 65 GHz. To ease the experiment, two fiber arrays (FA) are utilized to effectuate the coupling of the signal light from the fiber to the chip, and on‐chip DC pad and the DC connector on the printed circuit board (PCB) are interconnected through wire bonding.

Prior to system‐level experiments, each packaged chip undergoes a fundamental testing and calibration procedure. The baseline characterization includes not only measuring the optical insertion loss but also calibrating the functionality of each MZI optical switch. This involves applying electrical power to the corresponding phase shifter, testing each MZI switch, and recording the results to establish a lookup table for its operational states. During calibration, the process proceeds sequentially from the back to the front stages. By adjusting the optical switch in the preceding stage, the MZI under test is supplied with only one optical input path, allowing its operating state to be determined based on the output optical power. For each MRR unit in four channels, the other MZI switches are first set to the through state to isolate the MRR from their influence. The spectral response of each channel's MRR is then scanned by tuning its phase shifter, and the corresponding electrical power settings are recorded. Finally, a Gaussian pulse is injected into each channel, and fine adjustments are made based on the output waveform to ensure it aligns with theoretical simulations—confirming that the MRR operates in the differentiation mode. The corresponding PD current is recorded and serves as the initial value for the FPGA‐based real‐time error‐correction algorithm during the experiment. Insertion loss imbalances between channels are equalized using external optical attenuators.

### 5.2 FPGA Processing Speed Evaluation

Here, we evaluate the upper limit of the processing speed for the FPGA‐based real‐time error‐correction system. The on‐chip high‐speed photodetector features a 3‐dB bandwidth exceeding 5 MHz, introducing a delay (<200 ns) into the feedback loop. Besides, the ALINX Artix‐7 FPGA operates at a clock frequency of 200 MHz. The gradient descent algorithm, involving only basic floating‐point arithmetic, requires less than 1 μs to compute the voltage updates, which is virtually instantaneous on the time scale of thermal dynamics. Regarding the instrumentation, while the current implementation uses software‐timed Ethernet commands, the Keithley 2450 Source Meter is capable of digitizing rates exceeding several k/s when utilizing internal hardware triggering to eliminate communication stack overhead. Consequently, the ultimate speed limit of the correction mechanism is dictated by the thermal time constant τ of the on‐chip TiN heaters used for phase shifting. In standard 220 nm SOI platforms, the thermal transport dynamics typically result in a time constant of τ approx below 10 μs. Applying the relationship f=1/(2πτ), this corresponds to a physical 3 dB bandwidth of approximately 10–15 kHz. Therefore, by optimizing the peripheral interface to eliminate instrument communication latency, the current error correction architecture can theoretically span a bandwidth of up to 10 kHz, effectively suppressing faster environmental disturbances such as acoustic vibrations or mechanical resonances.

### 5.3 Experimental Setup and Data Analysis

In our experimental setup, a signal to be processed is generated using an AWG (Keysight M8199A) with a sampling rate of 128 GSa/s. This signal is then modulated onto an optical carrier using a commercial lithium niobate modulator(FTM7938EZ). Due to the grating coupler design employed for on‐chip optical I/O ports, a polarization controller (PC) is utilized to adjust the polarization of the input optical signal, thereby maximizing the on‐chip coupling efficiency. The output optical signal is first amplified by an erbium‐doped fiber amplifier (Amonics AEDFA‐PA‐35‐B‐F) and subsequently detected by an lnGaAs photodetector with a 70 GHz bandwidth. Finally, the waveform data are captured using a high‐speed real‐time oscilloscope (Keysight UXR0502A).

For demodulation of 5 × 25 Gbps WDM BPSK signals, a CW optical carrier generated by a laser is first fed into a ${\mathrm{LiNbO}}_{3}$ modulator. A sinusoidal signal with a frequency of 40 GHz, produced by AWG, is used to drive the modulator, generating a multi‐wavelength source comprising five distinct wavelengths. The optical spectrum of this frequency comb source is observed and recorded using an optical spectrum analyzer (OSA, YOGOKAWA AQ6370D). Subsequently, the multi‐wavelength source is amplified by an EDFA before being loaded into the photonic integrated circuit for further processing. Subsequently, the multi‐wavelength source is amplified by an EDFA before being loaded into the photonic integrated circuit for further processing. Subsequently, the demodulated multi‐wavelength signals are routed through a wavelength‐selective switch (II‐VI WS‐01000A‐C‐S‐1‐AA‐00) to achieve wavelength‐division multiplexing. The waveform of each wavelength‐specific demodulated signal is individually recorded and processed using digital signal processing (DSP) techniques to complete the demodulation process. Finally, the BER curves are measured to evaluate the system's performance under varying signal conditions.

## Author Contributions

Z. Z. C. conceived the concept of reliable general‐purpose analog computing and created the concrete on‐chip processor unit architecture. T. Z. designed the basic silicon photonic element and completed the circuit layout. T. Z. and S. C. Z. performed the basic measurements of the chip. S. C. Z., X. W. X. and S. G. H. led the design for FPGA‐based real‐time error‐correction algorithm and implemented the codes. B. W. Z. designed the system experimental plan and developed the DSP algorithm for demonstrated applications, T. Z. and B. W. Z. carried out the high‐speed measurements with the help of Y. Z. P. and analyzed the data. J. Y. completed the PCB assembly. T. Z., B. W. Z. and K. R. L. prepared the manuscript with contributions from all authors. Z. Z. C. led the whole research, Z. Z. C., X. C. X. and S. H. Y. supervised the project. T. Z., B. W. Z. and S. C. Z. contributed equally to this work.

## Conflicts of Interest

The authors declare no conflict of interest.

## Supporting information


**Supporting File**: advs74724‐sup‐0001‐SuppMat.pdf.

## Data Availability

The data that support the findings ofthis study are available from the corresponding author upon reasonable request.
