# Supplementary material for: Spatial‐Wavelength Multiplexing Error‐Controlled Photonic Analog Computing System
Source: Adv Sci (Weinh). 2026 Mar 13;13(28):e15689. doi: 10.1002/advs.202515689 (PMC13185880; doi:10.1002/advs.202515689)
Supplement: Supplementary file 1 — Supporting File: advs74724‐sup‐0001‐SuppMat.pdf. [file ADVS-13-e15689-s001.pdf]

Supplementary Materials for
Spatial-Wavelength Multiplexing Error-controlled Photonic Analog Computing System

Tao Zhu[1,2†], Bowen Zhu[1†], Shicheng Zhang[3†], Keren Li[4], Xianchen Wu[3],Yazhi Pi[1], Jie Yan[5], Daigao Chen[5], Bingli Guo[3], Xi Xiao[1,5], Lei Wang[1],Xiaochuan Xu[1,2*], Xuwei Xue[3*], Shanguo Huang[3*], Zizheng Cao[6*], Shaohua Yu[1*]

[1]Pengcheng Laboratory, ShenZhen, 518055, China.

[2]School of Integrated Circuits, Harbin Institute of Technology(Shenzhen), Shenzhen, 518055, China.

[3]School of Electronic Engineering, Beijing University of Posts and Telecommunications, Beijing, 100876,China.

[4]College of Physics and Optoelectronic Engineering, Shenzhen University, Shenzhen, 518055, China.

[5]National Optoelectronics Innovation Center, Wuhan, 430074, China.

[6]College of Integrated Circuits, Zhejiang University, Hangzhou, 311200, China

[*]Corresponding author. Email: xuxiaochuan@hit.edu.cn, x.xue@bupt.edu.cn, shghuang@bupt.edu.cn, zcaozju@zju.edu.cn, yush@cae.cn

[†]These authors contributed equally to this work.

This file includes:

Materials and Methods

Supplementary Text

Figures S1 to S10

Tables S1 to S2

Reference

Materials and Methods

Principle of differential based ODE solver

More details are presented here for principle of differential-based ODE solver. An optical temporal differentiator computes the first derivative of the optical field in the time domain, exhibits a transfer function of the form $T_\omega = j(\omega - \omega_0)$ in the frequency domain, where ω_0 represents the optical carrier frequency. This transfer function indicates that the transmission of the differentiator is linearly proportional to the frequency detuning from the central frequency, while the phase response undergoes an exact π-phase shift across the central frequency. The core processing unit in Fig. 1(c) can be reconfigured by adjusting the phase shifter (PS) to form a MRR coupled to a single straight waveguide. This reconfigurable ability enables precise control over the optical signal processing capabilities of the unit, making it a versatile component for applications requiring tailored spectral and phase responses. The transfer function of this configuration can be expressed as:

$$T(\omega) = \frac{S_0}{S_i} = \frac{j(\omega - \omega_0) + \frac{1}{\tau_i} - \frac{1}{\tau_e}}{j(\omega - \omega_0) + \frac{1}{\tau_i} + \frac{1}{\tau_e}} \tag{S1}$$

where ω_0 represents the resonance frequency, $1/\tau_i$ represents the power decay rate due to the intrinsic loss, $1/\tau_e$ represents the power coupling to the waveguide. Thus the reciprocal of photon lifetime can be expressed as $1/\tau = 1/\tau_i + 1/\tau_e$. Under the condition that the frequency detuning is significantly smaller than the 3-dB bandwidth of the resonator, the expression can be approximated as follows:

$$T(\omega) = j\tau(\omega - \omega_0) + \frac{\frac{1}{\tau_i} - \frac{1}{\tau_e}}{\frac{1}{\tau_i} + \frac{1}{\tau_e}} \tag{S2}$$

Under this condition, the MRR coupled to a single straight waveguide can be modeled as a temporal differentiator with certain gain plus a constant-output. In particular, when the microring resonator works in the critical coupling region $\tau_i = \tau_e$, we obtain $T_\omega = j\tau(\omega - \omega_0)$, which is a typical function for a first-order temporal differentiator.

The ODE solver configuration based on temporal differentiator presented in Fig. 3(a) can be achieved by spatial multiplexing utilizing MZI optical switches. The constant-coefficient first-order linear ordinary differential equation can be expressed as:

$$\frac{\mathrm{d}y(t)}{\mathrm{d}t} + x(t) = ky(t), \tag{S3}$$

where $x(t)$ represents the input signal, $y(t)$ is the equation solution (output signal) and k represents a positive constant of an arbitrary value. The equation can be numerically transformed to spectral domain using Fourier transformation:

$$Y(\omega) = \frac{1}{k - j\omega} X(\omega) \tag{S4}$$

In time domain, the equation can be expressed as:

$$y(t) = u(-t)e^{kt} \otimes x(t) \tag{S5}$$

The equation also can be expanded using Taylor expansion as follow:

$$Y(\omega) = \frac{1}{k - j\omega} X(\omega) = \frac{X(\omega)}{k} \sum_{n=0}^{\infty} \left(\frac{j\omega}{k}\right)^n \tag{S6}$$

Therefore, the time-domain representation of the equation can be derived by applying the inverse Fourier transform:

$$y(t) = \frac{1}{k} \sum_{n=0}^{\infty} (\frac{1}{k})^n \frac{d^n x(t)}{dt^n} \tag{S7}$$

It can be seen from the formula that the equation converges only when $|j\omega/k| < 1$. In fact, during the experimental process, there is a threshold for k. When this threshold is exceeded, a solution to the ODE cannot be obtained.

The operational principle of the ODE solver depicted in the Fig. 3(a) is as follows: In the first cycle, only the input signal $x(t)$ propagates within the loop. Consequently, $y(t)$ should be expressed as:

$$y_1(t) = \frac{1}{k} x(t) \tag{S8}$$

for the second circle, $y(t)$ should be expressed as:

$$y_2(t) = \frac{1}{k}(x(t) + \frac{dy_1(t)}{dt}) = \frac{1}{k}(x(t) + \frac{1}{k}\frac{dx(t)}{dt}) \tag{S9}$$

When the loop eventually reaches a stable state, the output expression can be written as:

$$y_n(t) = \frac{1}{k}(x(t) + \frac{dy_{n-1}(t)}{dt}) = \frac{1}{k}(x(t) + \frac{1}{k}\frac{dx(t)}{dt} + \cdots + (\frac{1}{k})^{n-1}\frac{d^{n-1}x(t)}{dt^{n-1}}) \tag{S10}$$

The derived expression is consistent with the Taylor series expansion presented earlier, representing the solution to the ODE under the input $x(t)$. This alignment confirms the mathematical validity of the approximation and its applicability to modeling the system's response to time-varying inputs. For higher - order ODE equations, it only requires taking the solution $y_1(t)$ of the first ODE as the input $x_2(t)$ of the second solver. The principle of each solver is the same.

Principle of BPSK signal receiving using MRR

More details are presented here for principle of BPSK signal receiving using MRR. Figure S1 presents the time-domain waveform of an original BPSK signal, along with the waveform after MRR-based differential transformation and PD detection from left to right. The corresponding spectral are shown below the waveforms. It can be observed that after differentiation, the BPSK waveform primarily consists of impulse-like functions at symbol transitions, with amplitudes depending on the direction of bit changes. This type of signal is also referred to as an alternate-mark inversion (AMI) signal. Since differentiation can be regarded as a notch filter, the AMI signal exhibits a notch at the spectral zero point. After passing through the PD, all positive and negative pulses are converted into positive values. By sampling at the correct timing, the original BPSK signal can be recovered through differential demodulation.

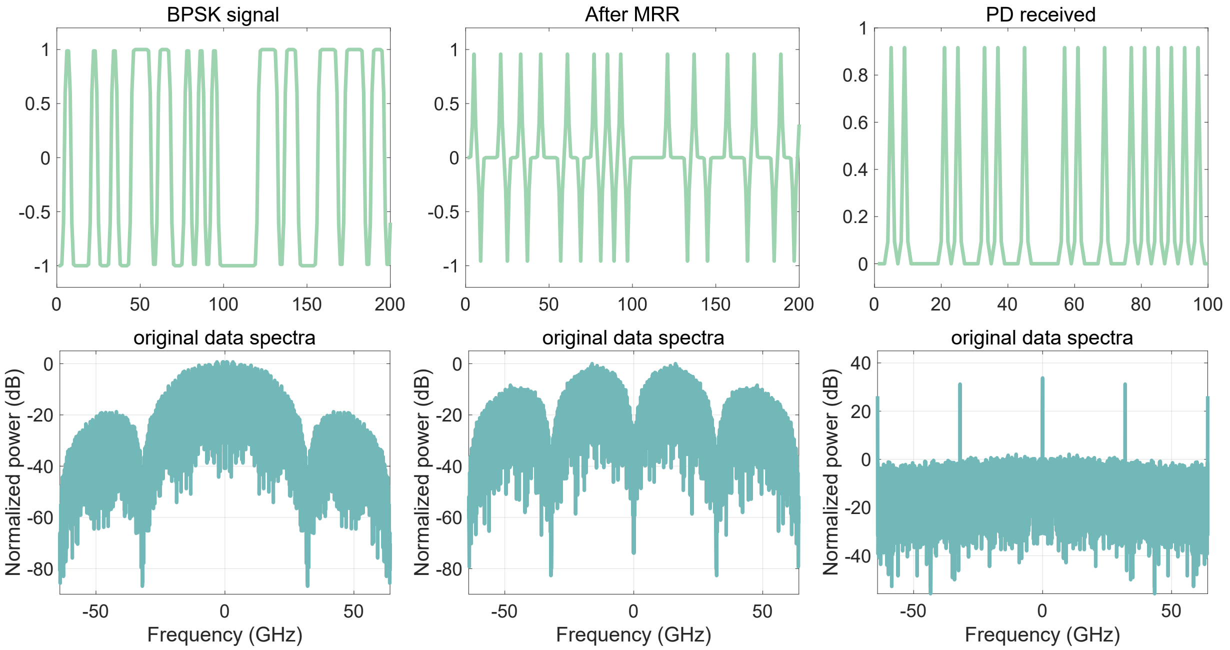

Figure S1: **Simulation results of BPSK signals demodulation using MRR.** The upper panels depict the temporal waveforms and the lower panels present their corresponding spectral profiles.

Supplementary Text

Signal-processing Accuracy Analysis

Here we investigate the factors within the system that influence signal-processing accuracy. It includes the components or devices in the optical link and the photonic chip itself. Signal fluctuations may occur in the external link of the system, primarily during the electro-optic conversion process. The operating point of the MZM is susceptible to temperature variations, which can cause drift and result in optical signal distortion. However, in practical applications, such as optical fiber communications, feedback control modules for the bias point of modulators have been well developed and are commonly used to mitigate these effects.

Large dispersion can cause pulse broadening or optical signal distortion, especially for signals exceeding 100 Gbaud, which limits the transmission distance of single-mode fiber to under 2 km. The waveguide lengths in on-chip processing are 3-4 orders of magnitude shorter than those in fiber. Therefore, the dispersion in silicon photonic chips can be safely ignored in the current experimental setup.

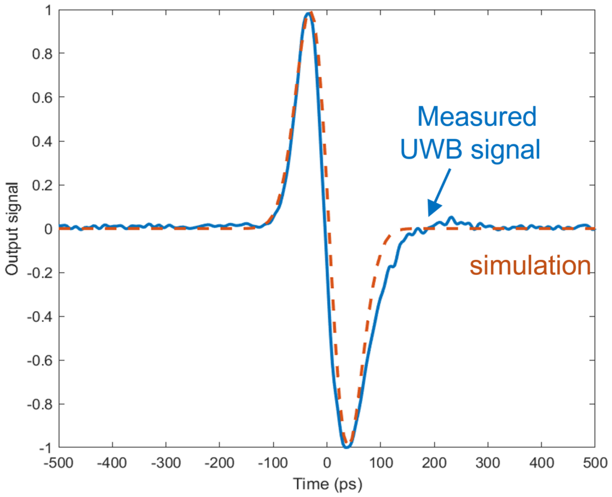

Figure S2: **Measured generated UWB signal.**

Additional, the bandwidth of the electronic equipment also affects the quality of the acquired signals. For instance, in the experiment where the chip is used to generate UWB signals, noticeable jitter appears in the trailing edge of the captured waveform, as shown in Figure.S2. This is because the oscilloscope in the measurement setup cannot accurately capture the high-frequency components

of the output signal, leading to distortion in the time domain. Therefore, electronic components in the system also influence the final processing results.

Crosstalk Analysis

Crosstalk is an inherent challenge in photonic integrated circuits and can degrade system's performance. In the present device, several physical mechanisms may contribute to crosstalk, including optical coupling crosstalk, thermal crosstalk between thermo-optic elements, electrical coupling between heater drivers, and nonlinear interactions. Among these, optical and thermal crosstalk are the most relevant for the system reported here.

Thermal crosstalk originates from the cumulative heat dissipated by the dense array of on-chip thermo-optic phase shifters. To address this, the FPGA-based real-time correction loop in our system is specifically designed to compensate for such temperature-induced resonance shifts and maintain stable operation over extended periods. Optical coupling crosstalk mainly comes from the finite extinction ratio of the MZI-based optical switches, which may lead to partial power leakage between channels. The limited extinction ratio of an on-chip MZI optical switch is primarily caused by imbalances in the power splitting/combining of its multimode interference (MMI) couplers and phase errors in its waveguide arms, stemming from fabrication-induced dimensional non-uniformities. We experimentally measured the inter-channel crosstalk by injecting optical power into each of the four channels individually and monitoring the output spectra at all ports, as shown in Figure.S3. The measured isolation >15 dB near 1550 nm. The residual response observed in other channels represents the combined effect of multiple cascaded processing units and switching elements.

Thermal crosstalk arises from the heat generated by the thermo-optic phase shifters integrated within the processing units. While thermal isolation trenches are incorporated around the heaters, long-term measurements indicate that residual thermal drift and slow thermal diffusion can still affect the resonance conditions of nearby microring resonators. This thermal interaction can alter the effective detuning or coupling conditions of the rings. To maintain stable operation, the system employs an FPGA-based real-time correction loop that continuously monitors on-chip photodiode readings and compensates for any thermally induced deviations from the desired operating point. Electrical coupling and nonlinear crosstalk are expected to be negligible in the present design. The

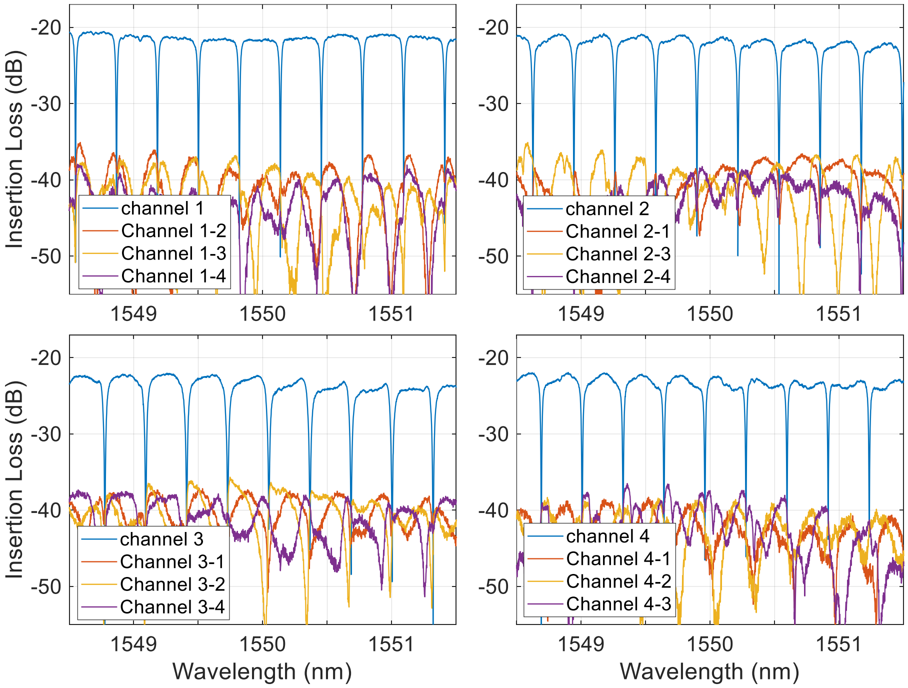

Figure S3: **Measured crosstalk of the four-channels.**

chip does not incorporate high-speed electrical modulation, and the optical power levels used in the experiments remain well below the threshold where nonlinear effects would become significant.

A combination of design, layout, and system-level approaches is used to keep crosstalk within acceptable limits. Increasing waveguide spacing or tailoring adjacent waveguide geometries can reduce unwanted optical coupling at the device level. At the layout level, careful placement of active components helps reduce thermal interaction. From a packaging perspective, TEC-based temperature control and stable thermal anchoring can suppress long-term temperature drift. Additionally, software-level compensation, including the real-time feedback control demonstrated in this work, provides an effective method to stabilize device operation even in the presence of slow thermal perturbations. Overall, although crosstalk imposes practical limitations on photonic signal processors, the combined strategies implemented in this work ensure that crosstalk does not significantly impair the demonstrated system performance.

Noise Accumulation Analysis

When an EDFA is inserted into a loop, the amplified output is partially returned to the gain medium, forming an effective cavity. Oscillation may occur if the net loop gain >1 and the loop phase approaches an integer multiple of 2π (Barkhausen criterion). More generally, even when the static loop gain is below unity, the intrinsic carrier-photon dynamics of the EDFA can cause under-damped relaxation oscillations or delayed re-gain events, potentially leading to power or phase instabilities.

A single-stage EDFA generates ASE within its gain band, and its spectral power density can be approximated as (with different factors for single-or dual-polarization cases):

$$S_{ASE}(\upsilon) = n_{sp} h\upsilon (G - 1) \tag{S11}$$

where n_{sp} is the spontaneous emission factor (equivalent noise factor), G is the linear gain, and h is the photon energy. If the net equivalent loop gain (including amplification and losses) in a certain frequency band satisfies $G_{loop} \geq 1$, the ASE will be amplified on each round-trip and may grow geometrically until limited by saturation or nonlinear effects.

Experimental validation of the critical gain and ASE growth behavior is shown here. The detailed experimental scheme is described below. To more directly observe the noise impact of the EDFA within the loop, we used a signal analyzer (Keysight N9040B) to analyze the signal spectrum. In setup (a), the laser output was attenuated and directly received by a PD before being fed into the signal analyzer. This serves as the reference for the entire test, representing the system's noise floor. In setup (b), we modulated a 32 Gbaud BPSK signal using a modulator and directly input the modulated signal into the PD. The PD bandwidth is 30 GHz, and the signal analyzer bandwidth is 50 GHz, satisfying the reception conditions. Next, we input the modulated signal into the chip. The output from the chip was amplified by the EDFA and then received by the PD. The EDFA model we used is Amonics AEDFA-PA-35-B-F. The figure below shows the noise figure provided by the manufacturer, with specific specifications as follows: Optical Gain @ -10 dBm > 20 dB, and Noise Figure @ -10 dBm < 4.3 dB at 1550 nm, classifying it as a low-noise amplifier. This step was designed to evaluate the impact of EDFA amplification on the signal. Finally, following the configuration described in the manuscript, we placed the same EDFA within a feedback loop to assess its effect in the system. Throughout the evaluation, the optical power input to the PD was

kept constant, and the SA video bandwidth was set to 1 kHz.

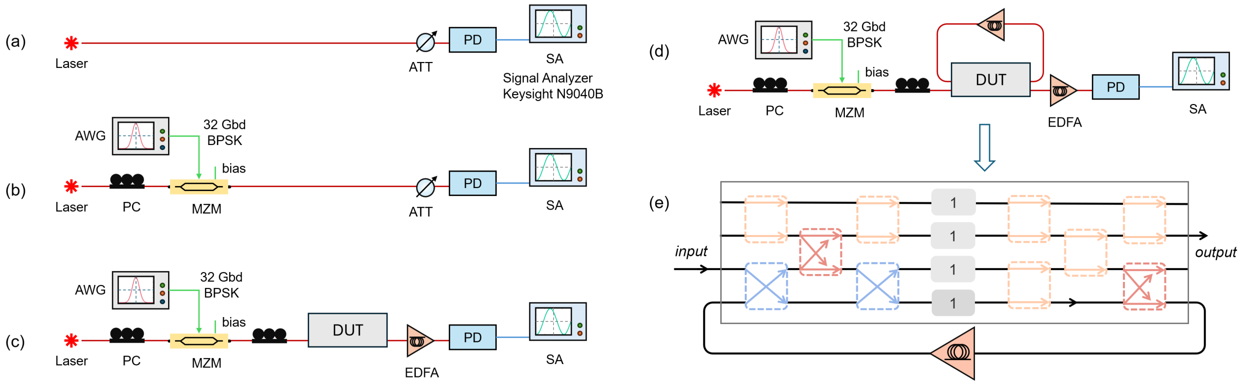

Figure S4: **Experimental setup of EDFA loop analysis.**

The test results are shown in the figure S5, first presenting the outcomes of setups (a) to (c). The green curve represents the result from (a) as the reference, indicating the noise level of the link system, including laser noise and PD response. The orange curve is the spectrum of the modulated signal directly received by the PD. Since the modulation rate is 32 Gbaud, the single-sideband bandwidth in the spectrum is about 16 GHz, with a signal-to-noise ratio (SNR) of approximately 20 dB. From the spectrum, it can be observed that the EDFA amplification does introduce a slight spectral elevation, with an increase of about 2 dB around 20 GHz. The test result for the EDFA within the feedback loop is shown by the purple curve in the following figure. From the test results, no significant signal oscillation or nonlinear effects were observed in the signal spectrum. However, signal quality degraded: the noise floor in the 20-30 GHz range increased by about 5 dB, and the SNR decreased to around 14 dB. The test result for the EDFA within the feedback loop is shown by the purple curve in the following figure. When placing an optical amplifier within a loop can introduce three classes of risks: gain-induced oscillation, ASE buildup, and nonlinear distortions. From the test results, no significant signal oscillation or nonlinear effects were observed in the signal spectrum. However, signal quality degraded: the noise floor in the 20-30 GHz range increased by about 5 dB, and the SNR decreased to around 14 dB. Noise accumulation and signal degradation were observed as the number of stages increased, indicating that further improvement in on-chip loss performance is necessary. These experiments directly address the dominant physical constraints that would affect deeper cascaded operation, such as noise buildup and stability, and provide experimental validation of the system's operating boundaries.

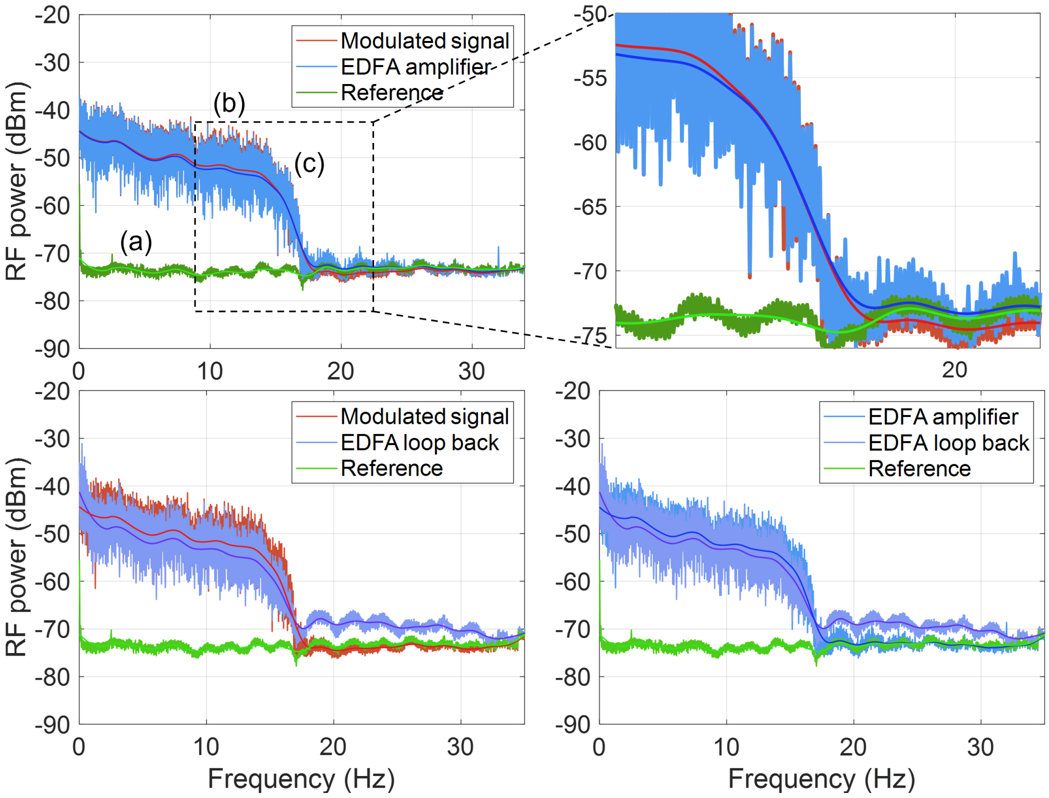

Figure S5: **Experimental results of EDFA noise accumulation analysis.**

Higher-order ODE solver investigation

Here, we investigate higher order ODE solving based on cascading multiple first-order ODE solver stages. Due to the current experimental constraint that only one single packaged chip is available, We adopted an equivalent cascading approach based on the theoretical decomposition of an Nth-order ODE into a cascade of first-order ODE solvers. In this scheme, the output waveform of a first-order ODE solver is recorded and then re-injected into the same photonic solver via an AWG, serving as the input to the subsequent solver stage. Experimentally, this is realized by using an AWG to generate the input waveform corresponding to the solution of the preceding order.

We acknowledge that this equivalent cascading scheme introduces additional optical-electrical-optical (O/E/O) conversions, which inevitably add electrical noise and DAC quantization noise. As a result, the experimental conditions are in fact more stringent than those of a fully integrated chip-to-chip cascading scenario. Nevertheless, this approach allows us to closely reproduce the functional behavior of cascaded solver stages and to directly observe noise accumulation across multiple

orders in a controlled manner. The equivalent cascading experiment provides a conservative yet effective verification of scalability, as any fully integrated cascading implementation would benefit from reduced O/E/O noise and improved signal fidelity.

Using this method, we experimentally demonstrate ODE solving up to the third order, including (i) first-order ODE solutions with two different coefficients, (ii) second-order ODE solutions with identical coefficients, (iii) second-order ODE solutions with unequal coefficients, and (iv) third-order ODE solutions.

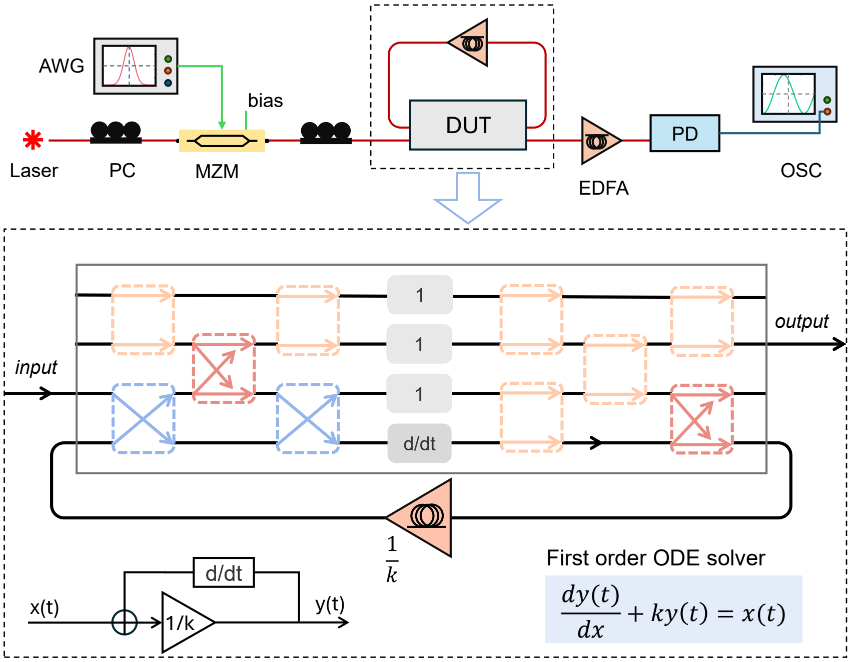

Figure S6: **Schematic of experimental setup for ODE solving.**

The system-level experimental setup is illustrated in Figure. S6. An arbitrary waveform generator (AWG) is used to generate the input signal, which is modulated onto an optical carrier using a Mach–Zehnder modulator (MZM). The modulated optical signal is then injected into the photonic integrated circuit (PIC). The on-chip configuration follows the same architecture described in the manuscript, where a fiber-based loop-back path incorporating an EDFA is employed to implement and tune the ODE coefficient k. After on-chip processing, the output optical signal is amplified by an EDFA and detected by a photodetector (PD). The time-domain result is captured using a high-speed oscilloscope (OSC).

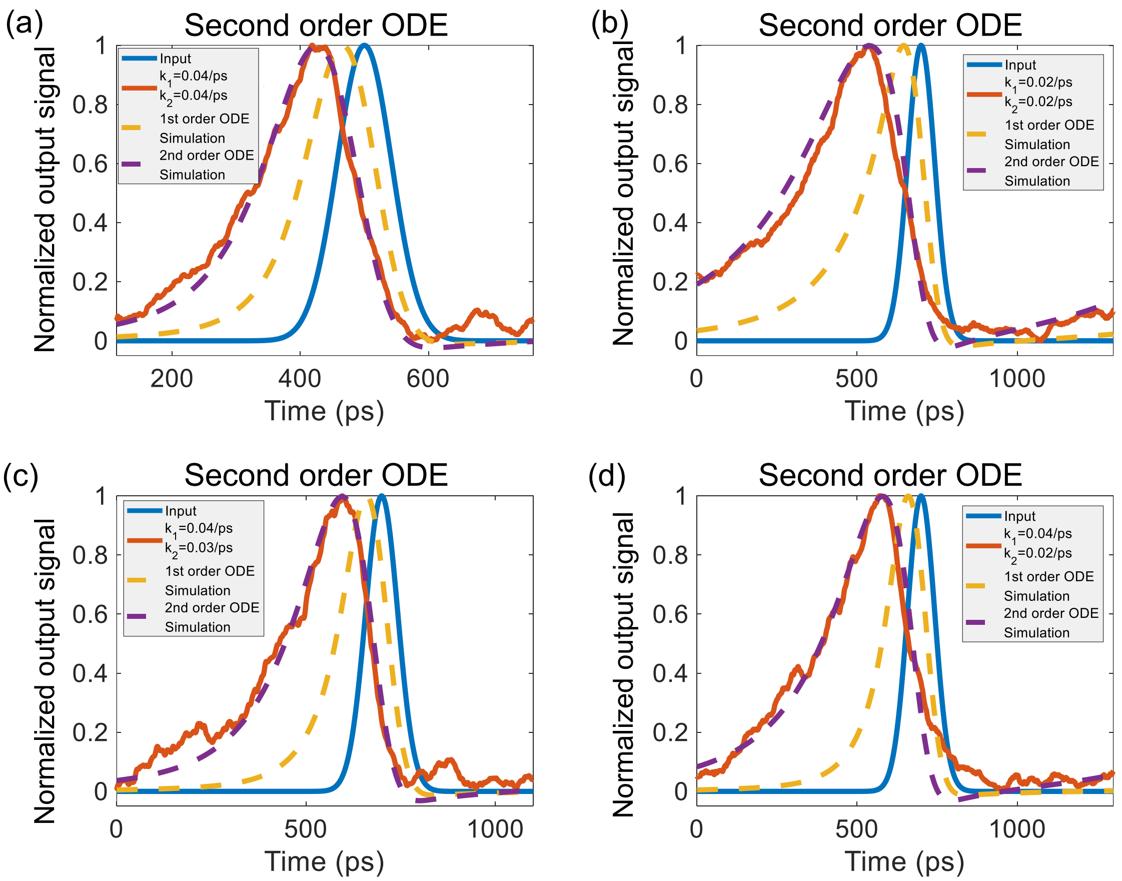

Figure S7: **Measured second-order ODE solutions.**

For the second-order ODE solving, the experimentally obtained waveform from the first-order ODE solver is first captured by the oscilloscope and then re-generated by the AWG. This waveform is subsequently modulated and re-injected into the photonic chip as the input to the second-stage ODE solver. The corresponding experimental results are shown in Figure. S7. In the second-order ODE formulation, the first-order coefficient is given by $p = 1/k_1$ and the second-order coefficient by $q = 1/k_2$. We first present the case where the first- and second-order coefficients are identical, as shown in Fig. S7(a) and (b). Compared to the first-order ODE results, the second-order output exhibits more pronounced waveform jitter and distortion. Nevertheless, the overall temporal evolution remains consistent with the theoretical and numerical simulations. The increased high-frequency components observed in the waveform are attributed to noise accumulation arising from the two-stage iterative computation and external electrical noise. In addition, we demonstrate the second-order ODE solving results with unequal first- and second-order coefficients, as shown in

Fig. S7(c) and (d), further confirming the flexibility of the proposed cascading approach.

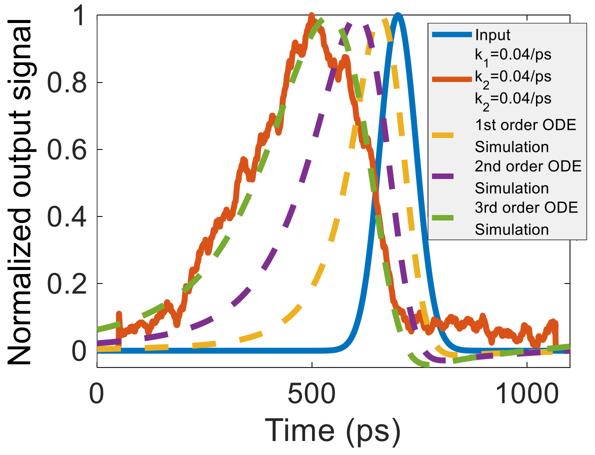

Figure S8: **Measured third-order ODE solutions.**

Finally, we extended the equivalent cascading scheme to third-order ODE solving, and the corresponding experimental results are shown in Figure. S8. The measured waveforms exhibit a significantly higher noise level compared to the second-order case, and the agreement with theoretical and numerical simulations is correspondingly reduced. This degradation is consistent with cumulative noise effects arising from multi-stage iterative processing.

This equivalent cascading strategy remains a valid verification of scalability because the proposed architecture is functionally modular: each first-order ODE solver constitutes a self-contained, reconfigurable computational primitive with fixed noise, loss, and transfer characteristics. This leads to a conservative and reproducible evaluation of cascade feasibility. Moreover, as the ODE order increases, the equivalent cascading process gradually transitions from an all-optical input to a hybrid analog-input regime. Specifically, the first-order ODE solver is driven by a purely optical input signal. To more closely approximate the practical cascading scenario, the output waveform of the first-order solver is first captured and then re-modulated onto an optical carrier, thereby preserving the noise characteristics inherent to the optical signal. This procedure ensures that the higher-order ODE inputs realistically reflect both signal distortions and noise accumulation present in cascaded operation.

Real-Time Error Correction for Photonic GPAC

Analog computing systems, including the photonic GPAC presented here, are inherently susceptible to environmental variations and component imperfections that lead to performance degradation. To ensure accurate and reliable computation, particularly for solving ODEs, the core processing unit, based on a reconfigurable micro-ring resonator, must be maintained in the critical coupling state, enabling accurate temporal differentiation. To achieve this, we have implemented an active, real-time error correction system based on a gradient descent algorithm, implemented on a field-programmable gate array (FPGA).

The primary source of error is the drift of the MRR's resonant frequency and operating point, mainly due to temperature variations, to which the thermal phase shifters are highly sensitive. Other contributing factors include laser power and wavelength fluctuations, amplifier gain drift, and photodetector responsivity changes. Error correction relies on the integrated on-chip photodetector (PD) at the drop port of the core processing unit. The PD current is directly proportional to the optical power at the drop port, providing a sensitive, real-time indicator of the MRR's resonance condition and its coupling state. Deviations from the maximum PD current, corresponding to critical coupling, signal a departure from the desired operating point.

The core processing unit, crucial for performing the differentiation operation in the GPAC's ODE solver, consists of a double-ring MRR coupled via Mach-Zehnder interferometers. The electric field at the drop port, E_d, can be described by the following transfer function:

$$E_d = \frac{-1j \cdot a_0 \cdot e^{j(\phi_1+\phi_2+\alpha+\gamma+\beta)/2} \cdot \cos(\alpha/2) \cdot \cos(\gamma/2) \cdot \cos(\beta/2) \cdot E_{in}}{D} \tag{S12}$$

where

$$\begin{aligned} D = {} & a_0^2 \cdot e^{j(\phi_1+\phi_2)} \cdot e^{j(\alpha+\gamma+2\beta)/2} \cdot \sin(\alpha/2) \cdot \sin(\gamma/2) \\ & - a_0 \cdot e^{j\phi_1} \cdot e^{j(\alpha+\beta)/2} \cdot \sin(\alpha/2) \cdot \sin(\beta/2) - a_0 \cdot e^{j\phi_2} \cdot e^{j(\gamma+\beta)/2} \cdot \sin(\gamma/2) \cdot \sin(\beta/2) + 1.0 \end{aligned}$$

where E_{in} is the input electric field. $a_0 = \exp(-\sigma L/2)$ is the round-trip transmission coefficient of a single MRR ring, with σ being the optical field transmission loss coefficient and L the single ring length. ϕ_1 and ϕ_2 are the phase shifts of light after one round trip in the two single rings, controlled by phase shifters PS4/PS5 and PS6/PS7, respectively. α, β, and γ are the phase differences between the two arms of the MZIs, controlled by phase shifters PS1, PS2, and PS3, respectively.

This complex transfer function highlights the multiple degrees of freedom available for controlling the MRR's operating state. For the MRR to function as a first-order differentiator, two key conditions must be met: 1) The MRR must be in the critical coupling state. 2) The resonant wavelength must match the input laser wavelength.

To simplify the control problem, we adopt the following strategy. PS2 is set to a fixed value, determining the free spectral range (FSR) of the core processor (either single-ring or double-ring configuration). PS6, which has a similar function to PS4 in controlling the MRR phase shift and resonant wavelength, is kept constant at 0V. PS1, which controls the input coupling ratio, is also set to a fixed value determined by the specific computational task. For the MRR to operate as a first-order differentiator, the through-port transfer function must exhibit a $\pm\pi/2$ phase shift near resonance, approximated as 'j' in the $j(\omega - \omega_0)$ term. This phase condition, derived from the full transfer function, dictates a specific relationship between the input and output coupling coefficients and is intrinsically linked to the round-trip phase accumulation within the ring. This leaves PS3, which controls the output coupling ratio, and PS4, which primarily affects the MRR phase shift and resonant wavelength, as the active control variables. Therefore, the combined optimization of PS3 and PS4 can meet both the critical coupling and resonant wavelength matching conditions of the MRR.

Figure S9 illustrates the dependence of the normalized drop port power on the voltages applied to PS3 (Va) and PS4 (Vb) for four different fixed settings of PS1 (represented by phase differences of -0.7π, -0.5π, 0.3π, and 0.4π between the MZI arms), with PS2 set to a 0.1π phase difference, selecting the double-ring configuration. These figures demonstrate two crucial points. First, the power landscape is smooth with respect to Va and Vb. This smoothness justifies the use of a gradient descent algorithm for optimization. Second, for each fixed PS1 setting, there exist periodic maximum power points, which can be reached by adjusting only PS3 and PS4. Therefore, by maximizing the PD current (proportional to the drop port power) using a gradient descent algorithm that adjusts Va and Vb, we can reliably bring the MRR to the critical coupling state and with the resonant wavelength matching the input laser wavelength, thus ensuring accurate first-order differentiation.

The gradient descent algorithm iteratively adjusts Va and Vb according to the following update equations:

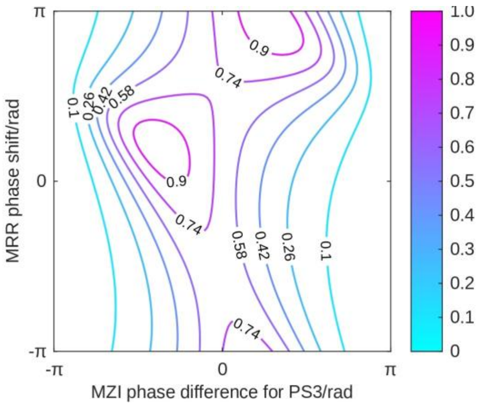

(a) PS1 @ 0.3π

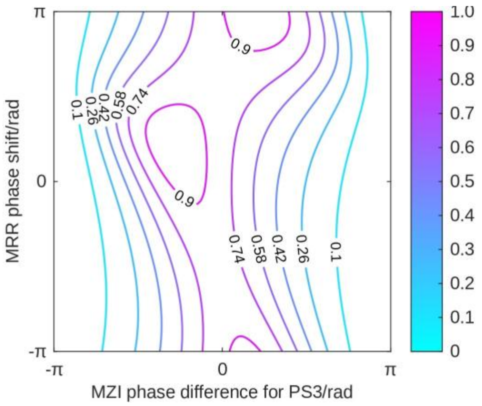

(b) PS1 @ 0.2π

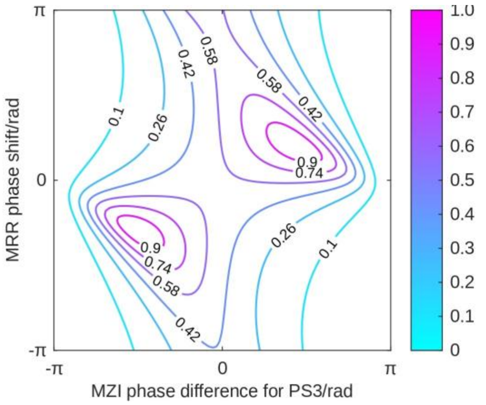

(c) PS1 @ -0.5π

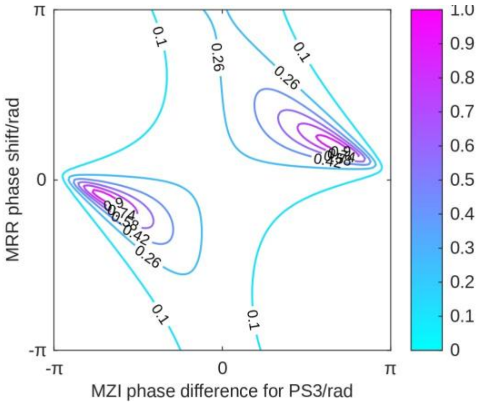

(d) PS1 @ -0.7π

Figure S9: **Normalized drop port power versus MZI phase difference (controlled by PS3 and PS4) for different fixed PS1 settings.**

$$V_{a\_new} = V_a - \eta \cdot \frac{\partial I}{\partial V_a} \tag{S13}$$

$$V_{b\_new} = V_b - \eta \cdot \frac{\partial I}{\partial V_b} \tag{S14}$$

where I is the PD current, and η is the learning rate. The partial derivatives are estimated numerically using finite differences:

$$\frac{\partial I}{\partial V_a} \approx \frac{I_{PD}(V_a + \Delta V_a, V_b) - I_{PD}(V_a, V_b)}{\Delta V_a} \tag{S15}$$

$$\frac{\partial I}{\partial V_b} \approx \frac{I_{PD}(V_a, V_b + \Delta V_b) - I_{PD}(V_a, V_b)}{\Delta V_b} \tag{S16}$$

where ΔV_a and ΔV_b are small voltage step sizes. An adaptive step size mechanism is employed, starting with $\Delta V_a = \Delta V_b = 0.02$ V and increasing by 0.01 V increments if the gradient magnitude, $Grad = \sqrt{(\partial I/\partial V_a)^2 + (\partial I/\partial V_b)^2}$, falls outside a predefined range ($Grad_{min}$ to $Grad_{max}$). This ensures that the gradient estimate is not dominated by noise (step size too small) or averaging effects (step size too large). The algorithm is triggered when the measured PD current deviates from its initial maximum value (obtained after the initial configuration of PS1-PS6) by more than a threshold value. The algorithm then iterates, adjusting Va and Vb, until the PD current is within 0.01 mA of its initial maximum, or until a maximum number of iterations (50) is reached. To mitigate the effects of high-frequency noise, the PD current, I_{PD}, used in the calculations, is the average of five consecutive readings from the Keithley 2450 source meter. Table 1 summarizes the key parameters of the algorithm. Figure S10 illustrates the flowchart of the real-time error correction algorithm for the photonic GPAC, which is based on gradient descent.

Table S1: **Gradient Descent Algorithm Parameters.** Here lists the parameters used in real-time correction algorithm and the optimization results of the gradient descent optimization.

Parameter	Symbol	Value	Justification
Initial Voltage Step Size (Va)	$\Delta V_{a\_initial}$	0.02 V	Balance between noise and accuracy
Initial Voltage Step Size (Vb)	$\Delta V_{b\_initial}$	0.02 V	Balance between noise and accuracy
Voltage Step Size Increment	$\Delta V_{increment}$	0.01 V	Adaptive step size adjustment
Learning Rate	η	0.05	Balance between speed and stability
Maximum Iterations	$Max\_Iterations$	50	Prevent infinite loops
PD Deviation Threshold	$Threshold$	0.01 mA	Target accuracy
Minimum Gradient Magnitude	$Grad_{min}$	0.1 mA/V	Avoid noise-dominated gradients
Maximum Gradient Magnitude	$Grad_{max}$	1 mA/V	Avoid averaging effects
Number of Averaged Samples	N_{avg}	5	Reduce high-frequency noise
Sampling Rate		50 Hz	Nyquist frequency consideration
Target Update Rate		≥ 5 Hz	Ensure the real time and rapid

The algorithm is implemented on an ALINX Xilinx Artix-7 CZ3452 FPGA, operating at a clock frequency of 200 MHz. A block diagram of the implementation is shown in Fig S10. The key modules include a Keithley 2450 interface for reading the PD current via Ethernet, a 128-channel voltage source control module utilizing a USB interface, a gradient calculation module

(implementing Equations S14 and S16 and averaging), a voltage update module, and a control logic module that manages the threshold checks and iteration count. The system utilizes floating-point arithmetic for ease of development, the gradient calculation and voltage update modules share a single floating-point divider and multiplier, as these operations are not performed concurrently.

The data flow begins with the FPGA receiving the averaged PD current from the Keithley 2450 via Ethernet. An integrated Ethernet MAC and custom protocol handler manage the communication, acquiring the averaged PD current data for the gradient calculation module.

The gradient calculation module is responsible for computing the partial derivatives $\frac{\partial I}{\partial V_a}$ and $\frac{\partial I}{\partial V_b}$ using the finite difference method. To mitigate the effects of quantization noise and improve the accuracy of the gradient estimation, a numerically stable implementation of the finite difference calculation is employed. This involves careful consideration of the order of operations and the use of appropriate scaling factors to prevent overflow or underflow in the floating-point calculations. The adaptive step size mechanism, controlled by the $Grad_{min}$ and $Grad_{max}$ thresholds, is implemented using a state machine within the control logic module.

The voltage update module implements the gradient descent update equations (Equations S14). The learning rate η is implemented as a fixed scaling factor applied to the calculated gradients. To ensure stability and prevent oscillations, the updated voltage values, V_{a_new} and V_{b_new}, are checked against predefined upper and lower bounds before being sent to the voltage source. This prevents the application of voltages outside the operational range of the phase shifters.

The 128-channel voltage source is controlled via a USB interface. The FPGA implements a USB device controller and custom protocol handler to manage communication and apply voltage updates to the selected channels. A double-buffering scheme ensures glitch-free transitions between voltage updates.

The control logic module orchestrates the entire error correction process. It implements the state machine that controls the sequence of operations, including reading the PD current, calculating the gradient, updating the voltages, checking for convergence, and managing the iteration count. The control logic also handles the adaptive step size mechanism and the threshold checks for both the PD current deviation and the gradient magnitude.

The control loop achieves an update rate of approximately 8 Hz, well within the target of at least 5 Hz, ensuring rapid compensation for drift. This update rate is limited primarily by the

communication and settling time of the 128-channel voltage source via USB (approximately 300 ms). The computation time within the FPGA itself is negligible in comparison.

To evaluate the performance of the real-time error correction system, experiments were conducted using a setup comprising the fabricated photonic GPAC chip, a Keithley 2450 source meter for high-precision PD current measurement, a 128-channel voltage source for controlling the phase shifters, and an ALINX Xilinx Artix-7 CZ3452 FPGA implementing the error correction algorithm. The GPAC was configured to perform a first-order differentiation of a Gaussian input pulse with a FWHM of 200 ps.

The effectiveness of the real-time error correction system was experimentally verified on the fabricated photonic GPAC chip, configured for first-order differentiation. As previously discussed and illustrated in Figure 4(b), the error correction algorithm successfully maintained the PD current within a narrow tolerance (RMS deviation of 0.005 mA) over a 30-minute period, representing an 89% improvement in stability compared to the uncorrected system (RMS deviation of 0.046 mA). This stabilization of the PD current, and thus the MRR operating point, ensured the accurate generation of the first-order derivative waveform (Figure 4(c)), demonstrating the system's ability to mitigate the effects of environmental drift and maintain reliable analog computation.

Practical Processing Capabilities Evaluation of Packaged Chip

The 2.725 TOPS/MRR calculation in the original manuscript represents only a theoretical upper bound. Here we provide a physically grounded processing capability evaluation that accounts for WDM scalability, multi-wavelength source requirements, optical loss accumulation, and crosstalk constraints. First, regarding multi-wavelength sources, advanced photonic sources such as Kerr micro-combs and electro-optic frequency-comb generators can provide dense spectral lines spanning the entire C-band. These sources have been experimentally shown to produce tens to hundreds of wavelengths with stable power levels, making them suitable for supplying large-scale WDM inputs to the processor. Therefore, the availability of dense multi-wavelength sources is not a limiting factor for the proposed architecture. Second, we have experimentally verified that the reconfigurable MRR processing units exhibit highly uniform spectral responses across the full C-band (1530–1565 nm). With an FSR of ~ 40 GHz, approximately 110 usable WDM channels are available, each exhibiting >20 dB extinction ratio. These measurements confirm that the device design supports

large-scale WDM operation in principle.

The actual achievable performance is constrained primarily by optical loss accumulation and power-handling limits. Here, we provide an estimated achievable processing capabilities of the packaged chip. Taking BPSK signal demodulation as an example, when performing a 32 Gbaud single-wavelength demodulation, only one core processing unit on the chip is in the working state. Under these conditions, the signal processing speed reaches 32 giga-operations per second (GOPS). When the GPAC photonic chip performs demodulation of WDM BPSK signals by the same one MRR, it does not increase the power consumption of the chip. For instance, in the experiment validating the demodulation of 5×25 Gbps WDM BPSK signals, the processing speed of the chip in this experiment is 125 GOPS. The measured BER shows HD-FEC sensitivities of -24 to -26 dBm for these five channels.

Scaling to larger numbers of channels requires accounting for accumulated optical loss. For example, if the same on-chip input optical power is maintained and the system is extended to 50 wavelength channels, the receiver sensitivity would theoretically need to decrease by more than 10 dB due to optical loss accumulation, i.e., the input optical power to the PD should be > -14 dBm. Considering that the maximum input optical power the chip can withstand is about 16 dBm (beyond which there is a risk of grating damage), and given that the on-chip MRR is configured with an insertion loss of about 30 dB during processing, the output optical power would be around -14 dBm. This level is also used to estimate the HD-FEC receiver sensitivity for 50 wavelength channels. Hence, the achievable computing power of the packaged chip is approximately 1.25 TOPS per MRR, which is the estimated practical upper limit for this chip. In summary, while the design and response of the processing unit support the entire C-band bandwidth, the actual achievable computing power of this particular chip is reduced, which is an area that requires further improvement.

Energy consumption analysis of the GPAC system

While the chip-level efficiency is a critical benchmark, a holistic evaluation requires a system-level analysis that accounts for all auxiliary electronic and photonic components essential for operation. To provide a practical assessment of the GPAC system's overall energy efficiency, we have developed a comprehensive power consumption model[1–9]. This model considers the entire signal path, from

the light sources at the transmitter to the Photodetector (PD) at the receiver. The power consumption of each key component is calculated based on its physical operating principles and state-of-the-art (SOTA) performance parameters, aiming for an aggressive yet realistic estimation of a highly optimized system. A detailed breakdown of the power budget for a single channel is presented in Table S2.

Table S2: System-Level Power Consumption Budget and Parameters per Channel.

**Component**	**Power Model**	**Parameters and Assumptions**[1]	**Calculated Power (mW)**
Laser Source	$P_{elec} = P_{opt}/\eta_{eo}$	P_{opt}=0 dBm, Wall-plug efficiency $\eta_{eo} = 30\%$	3.33
Signal Generator (DAC)	$P_{drv} = R_{data}E_{bit,DAC}$	R_{data}=25 Gbps, energy/bit $E_{bit,DAC}$ =1.5 pJ/bit	37.5
Modulator Driver	$P_{DAC}{\approx}R_{data}k_{eff}C_{mod}V_{swing}^2$	k_{eff}=1.5, C_{mod}=150 fF, V_{swing} = 2 Vpp	22.5
GPAC chip	From experimental data	Fixed power for on-chip thermal phase shifters	12
EDFA[2]	P_{EDFA}=$(P_{out} - P_{in})/\eta_{PCE}$	Link loss=9 dB, P_{out}=0 dBm, P_{in}=-9 dBm, η_{PCE}=15%	5.83
Photodetector (PD)	P_{PD}=$V_{bias}I_{dark}$	Link loss=9 dB, V_{bias}=2 V, I_{dark}=10 nA	≈0
TIA	P_{TIA}=$R_{data}E_{bit,TIA}$	R_{data}=25 Gbps, energy/bit $E_{bit,TIA}$ =0.7 pJ/bit	17.5

[1] All calculations are based on a channel data rate R_{data} = 25Gbps. Parameters are selected to reflect SOTA, power-optimized components.

[2] Per-channel power budget for a multi-wavelength system with 9 dB total loss (including MUX/DEMUX).

The power models in Table S2 are derived from the fundamental physical and operational principles of each device. The electrical power of the laser source is governed by its wall-plug efficiency (η_{eo}), which represents the conversion ratio from input electrical power to usable output optical power (P_{opt}). For high-speed digital and mixed-signal components like the DAC, TIA, and DSP, consumption is benchmarked by their energy-per-bit (E_{bit}), a standard figure-of-merit that encapsulates the CMOS switching energy and architectural complexity required to process a

single bit of information; total power is thus the product of this figure and the data rate (R_{data}). The modulator driver's power model is more nuanced, approximating the energy needed to charge and discharge the modulator's capacitive load (C_{mod}) across its required voltage swing (V_{swing}) at the data rate. The term k_{eff} is a critical efficiency factor that accounts for the driver's internal circuit inefficiencies and biasing overhead beyond the ideal capacitive charging energy. For the optical amplifier, the EDFA's electrical power is proportional to the net optical power it generates-the difference between its output and input power ($(P_{out} - P_{in})$)-divided by its power conversion efficiency (η_{PCE}). Finally, the photodetector's static DC power dissipation is modeled from its bias voltage (V_{bias}) and its intrinsic dark current (I_{dark}), which is typically negligible for high-performance diodes.

Based on the per-component budget, the total system power consumption and corresponding energy efficiency were calculated across the different operational scales. For a single-wavelength (N=1) system, the total power consumption is 97.39 mW, yielding a system-level efficiency of 0.257 TOPS/W. When scaling to a five-channel WDM system (N=5), the total power becomes 408.66 mW with an efficiency of 0.306 TOPS/W. At the theoretical maximum capacity of 109 channels, the system is projected to consume approximately 8.47 W, resulting in a system efficiency of 0.322 TOPS/W.

This comprehensive system-level analysis successfully validates the exceptional parallel processing capabilities of the GPAC architecture, demonstrating a massive scaling in computational throughput from 32 GOPS to 2.725 TOPS. The results affirm that the photonic core can handle a vast increase in data channels with no additional power penalty, a distinct advantage of frequency-division multiplexing. The crucial insight from this analysis lies in the distribution of power across the system. It is evident that while the GPAC chip maintains a remarkably low and constant power draw of just 12 mW, the primary bottleneck for system-level energy efficiency has shifted to the peripheral electronics. The power consumed by the extensive array of electronic I/O (DACs, drivers, TIAs, DSPs) scales linearly with the number of channels, becoming the dominant factor in the overall power budget. This finding does not diminish the value of the GPAC's core efficiency; rather, it strategically directs the path for future research and development. To fully harness the potential of such massively parallel photonic processors, the next technological frontier is the deep, co-packaged integration of electronics and photonics. By engineering power-efficient electronic

I/O that can be co-integrated with the photonic die, the system-level efficiency can be brought into closer alignment with the extraordinary performance demonstrated at the chip level, paving the way for truly transformative, energy-efficient computing architectures.

Reference

1. Miller, David AB. Device requirements for optical interconnects to silicon chips. *Proceedings of the IEEE* **97**, 7(2009): 1166-1185.

2. Coldren, Larry A., Scott W. Corzine, and Milan L. Mashanovitch. Diode lasers and photonic integrated circuits. John Wiley & Sons, 2012.

3. Varshney, A. K., Singhal, G., & Nayak, J. High power lasers for directed energy applications: Developments and challenges. *Infrared Physics & Technology* **136**(2024): 105064.

4. Chang Chang, et al. Non-hermitian engineered low power thermo-optic silicon phase shifter. *2022 Conference on Lasers and Electro-Optics (CLEO)*. IEEE, 2022.

5. Ke Li, David Thomson, Shenghao Liu, Weiwei Zhang, Wei Cao, Callum Littlejohns, Xingzhao Yan et al. 112G baud sub pJ/bit integrated CMOS-silicon photonics transmitter. (2022).

6. Talkhoocheh, Arian Hashemi. Holistic Design in High-Speed Silicon Photonics and Low-Power Electronics Platforms. California Institute of Technology, 2023.

7. Chen, Kuan-Chang. Energy-Efficient Receiver Design for High-Speed Interconnects. California Institute of Technology, 2022.

8. Nilsson, Johan. Explicit analytic efficiency equation for saturated counter-pumped fiber amplifiers: Application to cladding-pumped erbium-doped fiber amplifiers. *Optics Communications* **579** (2025): 131578.

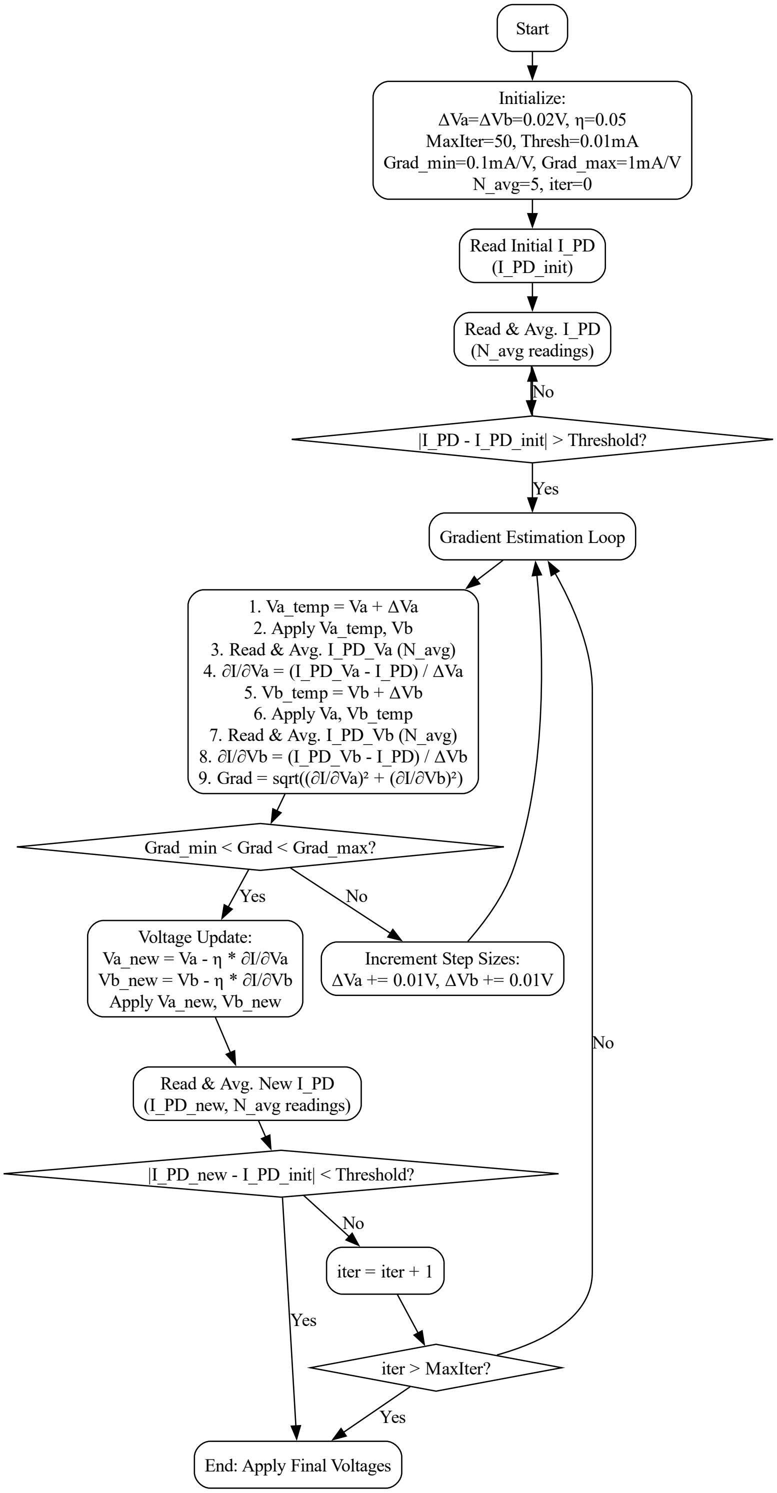

Figure S10: **Flowchart of the Real-Time Error Correction Algorithm Based on Gradient Descent.**
